# Mechanism and Kinetics of Non-Electroactive Chlorate Electroreduction via Catalytic Redox-Mediator Cycle Without Catalyst’s Addition (EC-Autocat Process)

**DOI:** 10.3390/molecules30163432

**Published:** 2025-08-20

**Authors:** Mikhail A. Vorotyntsev, Pavel A. Zader, Olga A. Goncharova, Dmitry V. Konev

**Affiliations:** 1 Frumkin Institute of Physical Chemistry and Electrochemistry Russian Academy of Sciences, Leninsky prospekt 31-4, Moscow 119071, Russia; goncharovaooolga@gmail.com (O.A.G.); dkfrvzh@yandex.ru (D.V.K.); 2 Federal Research Center for Problems of Chemical Physics and Medicinal Chemistry Russian Academy of Sciences, N.N. Semenov’s avenue 1, Chernogolovka 142432, Russia

**Keywords:** chlorate electroreduction, multi-electron process, acidic medium, potentiostatic electrolysis, redox-mediator cycle, electrochemical autocatalysis, spectrophotometric analysis, chlorine dioxide ClO_2_, chlorous acid HClO_2_

## Abstract

In the context of chlorate’s application as a cathodic reagent of power sources, the mechanism of its electroreduction has been studied in electrochemical cells under diffusion-limited current conditions with operando spectrophotometric analysis. Prior to electrolysis, the electrolyte is represented as an aqueous mixed NaClO_3_ + H_2_SO_4_ solution (both components being non-electroactive within the potential range under study), without addition of any external electroactive catalyst. In the course of potentiostatic electrolysis, both the cathodic current and the ClO_2_ concentration demonstrate a temporal evolution clearly pointing to an autocatalytic mechanism of the process (regions of quasi-exponential growth and of rapid diminution, separated by a narrow maximum). It has been substantiated that its kinetic mechanism includes only one electrochemical step (chlorine dioxide reduction), coupled with two chemical steps inside the solution phase: comproportionation of chlorate anion and chlorous acid, as well as chlorous acid disproportionation via two parallel routes. The corresponding set of kinetic equations for the concentrations of Cl-containing solute components (ClO_3_^−^, ClO_2_, HClO_2_, and Cl^−^) has been solved numerically in a dimensionless form. Optimal values of the kinetic parameters have been determined via a fitting procedure with the use of non-stationary experimental data for the ClO_2_ concentration and for the current, taking into account the available information from the literature on the parameters of the chlorous acid disproportionation process. Predictions of the proposed kinetic mechanism agree quantitatively with these experimental data for both quantities within the whole time range, including the three characteristic regions: rapid increase, vicinity of the maximum, and rapid decrease.

## 1. Introduction

In recent times, one can note a rapid increase in the number of publications devoted to the development of various electrochemical power sources, which are oriented to satisfy particular requirements regarding their size, specific and total energy and power, their costs, etc., as imposed by their prospective users. Besides the principal axes of activities in this research area (fuel cells and primary/rechargeable batteries with solid or solute electroactive components), much attention has been paid to a relatively novel direction: hybrid chemical power sources where one of the electrodes functions in a redox flow battery manner, i.e., its electrochemically active reagent is stored inside an external reservoir: in the course of functioning, the reagent is transported from its reservoir through the discharge device to generate or consume electricity in/from the external circuit. Owing to two different semi-elements of the discharge device, such hybrids combine the advantages of purely solid-state and flow-through liquid or gas systems, with the primary aim of reaching a higher energy density of the device (for electricity generation or/and energy storage). Since conventional redox couples such as metal/its solute ion or hydrogen/solute protons (hydroxonium cations) are among the leading anodic semi-elements with respect to their specific redox capacities (per 1 dm^3^ or per 1 kg of the reagent) and reversibility properties, they are mostly used in the negative electrode compartment. As for positive electrodes of hybrid power sources, various oxidants have been tested for various systems, in particular solutions of halogens [1,2,3], hydrogen peroxide [4,5], permanganates [6], chromates [7], salts of metal cations of variable oxidation degrees [8,9,10], as well as organic compounds [11].

For prospective applications in mobile devices, such hybrid devices should possess a much higher energy density. As a way to strongly increase this parameter, it has been proposed [12] that *multi-electron oxidizers* such as bromates and chlorates be used in aqueous acidic media as cathodic reagents, in combination with, e.g., hydrogen–oxidation reaction at anode in hybrid power sources. From a thermodynamic point of view, such a H_2_/LiBrO_3_ battery promises very high values in its redox-charge and energy densities, owing to a high salt solubility and potentially to its six-electron transformation to LiBr [13].

However, the bromate anion is *non-electroactive* within the suitable potential interval, i.e., it is *not* reduced with a measurable rate *at the electrode surface*, even at a specially modified one.

A conventional way to overcome this fundamental difficulty is to use the EC’ (or EC-cat) mechanism [14,15,16,17,18,19,20,21,22,23,24,25,26,27,28,29,30] where the principal (non-electroactive) oxidant, A, is transformed progressively on the basis of a redox couple, Ox/Red, via a mediator cycle where A is irreversibly reduced by Red into product P via a chemical reaction inside the solution phase as follows:

A + Red = P + Ox(1)

while Ox reacts *at the electrode*, with regeneration of the Red species as represented by

Ox + ne^−^ = Red (2)

Such a redox-mediator catalytic cycle, based on the combination of steps (1) and (2), has already found important practical applications [31], e.g., to prevent both Cl_2_ and O_2_ evolution reactions as well to reduce electric energy losses in the hydrogen production electrolysis from sea water. The redox couple, Ox/Red, may either be dissolved in solution or immobilized as a redox-active layer at the anode surface. If the couple in the solution is sufficiently *reversible electrochemically*, i.e., its components react in the vicinity of the standard potential of the couple, and this potential is *markedly lower* than the potential of the oxygen evolution process, the passage of the whole electrochemical reaction (including the H_2_ evolution) would require much lower electric energy, and the anodic process may be carried out at the electrode made of a cheap material. An alternative mechanism of the counter electrode reaction is based on a different variant of the redox-mediator cycle where Cl^−^ anion is electro-oxidized to Cl_2_, which is transformed into Cl^−^ and ClO^−^ under alkaline conditions; the latter species reacts with an organic molecule to synthesize a valuable product.

One should keep in mind that analyses of the kinetics of a reaction corresponding to this mechanism (1)–(2) studied under various experimental conditions showed that its rate is proportional to *the concentration of the catalytic species*, [Ox], *in the bulk solution* (Red is absent there in view of reaction (1)), one has *to add a sufficiently large amount of this catalyst* into solution of the principal reactant, A, in order to reach a sufficiently strong current. This feature of the EC-cat mechanism is evidently a serious disadvantage for its use in power sources based on reagent A since an extra reservoir is needed for the storage of the catalyst, i.e., Ox solution.

A breakthrough in this area was achieved in the studies of the bromate electroreduction in aqueous acidic medium. It was found theoretically [32] that this process could also proceed via a *mediator cycle*, which was based on the *Br_2_/Br^−^ redox couple*:

BrO_3_^−^ + 5 Br^−^ + 6 H^+^ = 3 Br_2_ + 3 H_2_O in solution, Br_2_ + 2 e^−^ = 2 Br^−^ at electrode surface (3)

This reactional scheme *looks quite analogous* to that for the EC-cat mechanism, Equations (1) and (2), except for the non-unity stoichiometric coefficients. However, it was discovered that the predictions for the current for scheme (3) are *crucially different* from those for the EC-cat mechanism. Namely, under conditions of *a moderate convection intensity* the passing current is restricted by the *diffusion-limited current for the principal reagent* (BrO_3_^−^) rather than by that *for the catalytic component* (Br_2_), *even if the Br_2_ concentration in the bulk solution is very small* (in the millimolar or even micromolar range) in the presence of the *molar-range* concentration of BrO_3_^−^_._

This drastic deviation from predictions for the EC-cat mechanism originates from an *autocatalytic* feature of mediator cycle (3) [13,32]: 5 Br^−^ anions entering its chemical step give 3 Br_2_ molecules which may *under advantageous conditions* produce 6 Br^−^ via the electrochemical step. This property is a direct consequence of a specific feature of scheme (3) that *both the principal reagent* (BrO_3_^−^) *and the catalytic redox couple* (Br_2_/Br^−^) *include the same chemical element*, Br.

As a result, a potential shift to the proper range induces the functioning of the redox cycle which leads to an *exponential increase* in the catalytic species, Br_2_ and Br^−^, near the electrode surface, accompanied by a parallel increase in the passing current [33]. This stage of the process terminates after the current’s approach to the (steady or non-stationary) diffusion-limited current *for bromate anion*.

Owing to this *unique* feature of the bromate process via scheme (3), there is *no necessity to add a catalyst* (Br_2_) *into the initial bromate-acid solution* since a small amount of Br_2_ (required for an “ignition” of cycle (3)) is generated “automatically” via the *chemical* BrO_3_^−^ decomposition in the course of the solution preparation. Then, owing to the current passage, the bromine concentration will grow exponentially *rapidly* up to its high amount, which is sufficient for a rapid BrO_3_^−^-to-Br^−^ transformation via global reaction (4):

BrO_3_^−^ + 6 H^+^ + 6 e^−^ = Br^−^ + 3 H_2_O (4)

In view of these completely different properties of the process described by scheme (3), it was concluded that it represents an example of a *novel electrochemical mechanism* denoted as EC” (or EC-autocat) [13,32,33], which is based on a *redox-mediator autocatalytic cycle*.

All these theoretical predictions on the possibility to reach very high power and energy densities *without catalyst’s addition* [13,32,33] were later confirmed by experimental studies of this process inside electrochemical cells [13,34]. Then, these findings paved a way to the conceptualization and home-made construction of very first functioning H_2_/BrO_3_^−^ batteries [35,36,37,38], which demonstrated decent characteristics: combination of high currents and specific powers with a high efficiency of the bromate-to-bromine transformation.

Thermodynamical parameters for the chlorate reduction process are even more promising (owing to their higher solubilities in water) than those for bromates so that even NaClO_3_ and Ca(ClO_3_)_2_ might be considered as prospective cathodic reagents in power sources. Additionally, chlorates are much cheaper than the corresponding bromates and they are easier available for industrial applications.

Absence of the electroactivity of ClO_3_^−^ anion, i.e., no *direct electrochemical* transformation of ClO_3_^−^ at an electrode surface, requires addressing again a *redox-mediator cycle*, preferably possessing *autocatalytic features*. An evident choice *would seem* to be to use the same EC-cat mechanism as that for the *bromate reduction*, scheme (3), replacing Br by Cl, i.e., scheme (5), based on the Cl_2_/Cl^−^ redox couple:

(5) $${{\mathrm{ClO}}_{3}}^{-}+\;5\;{\mathrm{Cl}}^{-}+\;6\;H^{+}\to \;3\;{\mathrm{Cl}}_{2}+\;3\;H_{2}O\;\mathrm{in}\;\mathrm{solution},\;{\mathrm{Cl}}_{2}+2e^{-}=2{\mathrm{Cl}}^{-}\mathrm{at}\;\mathrm{electrode}\;\mathrm{surface}$$

However, the available experimental data for the chemical stage, i.e., for the reaction between ClO_3_^−^ and Cl^−^ (see, e.g., [39]), show that its rate is *many decimal orders of magnitude slower* than that of the analogous chemical step for Br-containing components in scheme (3), which means *a very low rate of the whole process* for chlorates.

One should keep in mind that the mechanism of the reaction between chlorate and chloride turned out to be more complicated [39,40,41,42,43,44,45,46,47,48,49,50,51]: the process proceeds principally according to a scheme that leads to a mixture of two products, Cl_2_ and ClO_2_:

(6) $$2\;{{\mathrm{ClO}}_{3}}^{-}{+\;2\;\mathrm{Cl}}^{-}{+\;4\;H}^{+}\to {\mathrm{Cl}}_{2}{+\;2\;\mathrm{ClO}}_{2}{+\;2\;H}_{2}O$$

while the chemical stage of scheme (5) represents merely a side process. As a result, the reaction between ClO_3_^−^ and Cl^−^ gives a ratio of their yields: *r*(Cl_2_)/*r*(ClO_2_), which depends on the ratio of the initial concentrations: [Cl^−^]/[ClO_3_^−^]. This feature of the process represents an obstacle for its use for an industrial production of chlorine dioxide (ClO_2_) because of its contamination by Cl_2_.

As one can see below, chlorine dioxide plays a *key role* in the chlorate electroreduction process. In this context, it is worth mentioning that an alternative method for the ClO_2_ production [52,53,54,55] is based on the cathodic electrolysis of an acidized chlorate solution in the presence of solute ClO_2_. It is assumed that the product of the ClO_2_ electroreduction:

(7) $${\mathrm{ClO}}_{2}{+\;H}^{+}{+\;e}^{-}{=\;\mathrm{HClO}}_{2}$$

i.e., chlorous acid (HClO_2_), reacts with chlorate anion:

(8) $${\mathrm{HClO}}_{2}+\;{{\mathrm{ClO}}_{3}}^{-}{+\;H}^{+}{=\;2\;\mathrm{ClO}}_{2}{+\;H}_{2}O$$

generating an *extra* ClO_2_ molecule from ClO_3_^−^ anion. If this *excessive* chlorine dioxide is continuously removed from the cell, e.g., via its evaporation, the electrolysis process may be continued under steady-state conditions, except for a progressive diminution of the chlorate concentration.

Contrary to *predictions* of *very slow rates* of the chlorate reduction for *hypothetical* mechanism (5) (see above), our previous study of this process in acidic media inside an electrochemical cell [56] revealed *a much faster chlorate-to-chloride transformation* than it would be via scheme (5). These results provided us with a basis for design and home-made construction of the ever first power sources based on the chlorate-reduction process at the cathode [57,58]. These devices demonstrate prospective characteristics in terms of the faradaic efficiency of the chlorate-to-chloride transformation, maximal and average power densities, energy-transformation efficiency, etc. It means undoubtedly that the real process proceeds via *a different reactional scheme*, rather than scheme (5).

At the same time, experimental evidence [56,57,58] testifies directly in favor of the EC-autocat mechanism related to a *redox-mediator autocatalytic cycle* but based on a *different redox couple*.

A redox cycle composed of steps (7) and (8) *might* represent a potential candidate. This scheme allowed us to explain our experimental observations for the *chlorate electroreduction in strongly acidic medium* [56,57,58] that (analogous to the bromate electroreduction process, see above) there was *no necessity to add any catalyst inside such a solution*, despite the absence of any electroactivity of ClO_3_^−^ or acid at the electrode. Namely, a small amount of ClO_3_^−^ is *decomposed chemically in the course of preparation of its acidic solution*, similar to the partial BrO_3_^−^ decomposition in the course of its dissolution in an acidic solution, as it is evident from the solution’s spectra. Then, these generated ClO_2_ species participate in the *autocatalytic* redox-mediator cycle, Equations (7) and (8), leading to a progressive increase in both their concentration, [ClO_2_], and the current.

However, Schemes (7), (8) cannot explain *another key feature* of the chlorate reduction process since it predicts HClO_2_ *as its final product*, which means the transfer of *two electrons per ClO_3_^−^ anion*. A comparison of experimental data [57,58] for the total charge of the passed electricity with its upper limit corresponding to *the 6-electron process of the chlorate-to-chloride reduction*:

(9) $${{\mathrm{ClO}}_{3}}^{-}{+\;6\;H}^{+}{+\;6\;e}^{-}{=\;\mathrm{Cl}}^{-}{+\;3\;H}_{2}O$$

revealed that much more than two electrons per one chlorate ion were generated in the external circuit.

This implies that HClO_2_ molecules are also subject to *their further transformation* in the course of the chlorate reduction process. Since this step plays an important role in the course of the whole chlorate reduction process, its description on the basis of the available information [59,60,61,62,63,64,65,66,67,68,69,70,71,72,73,74] concerning properties of this species, especially concerning various HClO_2_ transformation schemes and expressions for the rate of this stage, is given in more detail below.

The objective of this study was to establish the principal stages of the mechanism of the global chlorate reduction in an acidic medium inside the electrochemical cell under the current passage. For this goal, the process was studied experimentally under chronoamperometric conditions for various amplitudes of the potential step as well as for various initial concentrations of NaClO_3_ and H_2_SO_4_. Results of a smaller-amplitude experiment were chosen for a detailed quantitative analysis owing to its simpler kinetic scheme (only one electrochemical stage). Synchronic temporal evolutions of the current and solution’s spectrum were registered. A reactional mechanism of the global process is proposed on the basis of the available experimental information. A set of kinetic equations for solute Cl-containing components is proposed for a description of the process. Then, numerical solutions of these equations were used for the *quantitative* interpretation of the experimental information for the evolution of the passing current, *I*, and chlorine dioxide concentration, [ClO_2_]. Such a procedure has allowed us to substantiate the proposed mechanism of both the global process and the HClO_2_ disproportionation step as well as to determine the principal parameters of the system.

These results will be of importance for future applications of this process for power sources.

## 2. Results and Discussion

### 2.1. Electrochemical Studies of Chlorate Reduction Process. Qualitative Discussion of Experimental Data

Chronoamperometric studies of this process have been performed under several different conditions. Measurements were carried out with the use of the experimental installation described in Section S1 of Supplementary Information: electrochemical cell with separated compartments for potentiostatic electrolysis of a known volume of aqueous sulfuric acid NaClO_3_ solution.

Three series of experiments were performed, with variation of the NaClO_3_ or H_2_SO_4_ concentration, or the amplitude of the potential step: see the three subsections below.

#### 2.1.1. Chronoamperometry: 8 M H_2_SO_4_; Potential Step to 0.1 V

**Figure 1:**
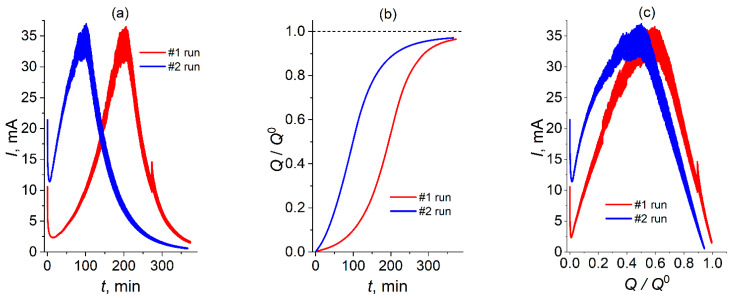
Chronoamperometric data for potential step from OCP to 0.1 V vs. Ag/AgCl (sat. KCl). Solution composition: 50 mM NaClO_3_ + 8 M H_2_SO_4_. Variation in current (**a**) or normalized passed reduction charge, *Q/Q*^0^, where *Q*^0^ = 6 *F V* [NaClO_3_] is the total reduction charge of the initial chlorate solution (**b**) as functions of time, *t*. Corresponding current vs. charge plots (**c**). Strong “noise” of the current in (**a**,**c**) as well as in subsequent figures originates from an *unsteady supply* of the electrolyte solution by the pump resulting in oscillations of the diffusion-layer thickness and consequently in rapid fluctuations of the current which are not resolved in (**a**,**c**) due to a very extended measurement time (over 5 h); see Section S1 “Materials and Methods” (inside Supplementary Information) for more details. Since the redox charge, *Q*, is found by integration of the current, *I*, over time, *t*, the fluctuations are practically absent in (**b**).

First, several chronoamperometric curves were measured for chlorate reduction in the aqueous 50 mM NaClO_3_ + 8 M H_2_SO_4_ solution under potentiostatic conditions: potential step to 0.1 V vs. Ag/AgCl (sat. KCl) (Figure 1a).

First of all, one should emphasize that the solution prior to each experiment was obtained by dissolution of a weighted amount of the solid NaClO_3_ powder in aqueous solution of sulfuric acid (as a source of protons), i.e., *without addition of any Cl-containing catalytic agent*. Nevertheless, the potential step resulted in *passage of a non-stationary current*, *I*(*t*) (Figure 1a), which started (after a conventional short-time relaxation) from a relatively low value of the current, followed by its *drastic increase* within an extended time range, with the subsequent passage through a *maximum* of the current and its *drastic decrease* to very small values after passage of the maximum.

This shape corresponds perfectly to theoretical predictions for the temporal variation of the current in the course of the *halogenate electroreduction* via *autocatalytic (EC-autocat) mechanism* [33] based on the hypothesis that a low initial concentration of the catalyst (e.g., molecular bromine in acidic bromate solution for the case of the *bromate reduction*) is *present in solution*.

The catalyst’s appearance inside the initial mixed solution is a consequence of a *partial chemical decomposition* of bromate or chlorate in the course of its dissolution in a sufficiently concentrated acid. The amount of the produced component (e.g., Br_2_ for the bromate process) depends on *uncontrollable* factors in the course of the mixed solution preparation. One may assume that a similar effect is also observed in Figure 1. As a result, the amount of the catalytic species for the chlorate reduction (discussed in detail below) varies obviously for different tests, as it follows from a much low intensity of the catalytic current within the starting range for the red line, compared to the blue one, in Figure 1.

A *unique* feature predicted [33] for the EC-autocat mechanism is the possibility for the current under potentiostatic conditions to reach *very high* values, even if the initial amount of the generated catalyst is *very low*. This theoretical prediction is confirmed by the data in Figure 1a: even though the initial values of the catalytic current for the red line are much lower than those for the blue one, the *maximal* values of the current are almost identical for these tests. At the same time, a lower initial value of the catalyst’s concentration for the red line results in a *much longer* duration of the current increase since its amount should be *accumulated in solution* due to the *autocatalytic* character of the whole process.

Integration of the current-vs.-time plots in Figure 1a gives the corresponding dependences of the passed charge, *Q*, on time. In Figure 1b, they are shown for the charges, *Q*, normalized by the *total reduction charge* of the initial chlorate solution calculated with the use of the formula *Q*^0^ = *n F V* [NaClO_3_], where *V* is the volume of solution, [NaClO_3_] is the initial concentration of chlorate, *F* is the Faraday constant, while *n* = 6 for the chlorate transformation into chloride, according to scheme (9).

One can see that both red and blue lines in Figure 1b approach a constant value (around 0.97) within the long-time range, as a consequence of the current’s approach to zero. Its proximity to 1 implies that the process terminates in conformity with reactional scheme (9) with 97% efficiency, independent of the initial amount of the generated catalyst (amount of decomposed chlorate in the course of the mixed solution preparation is negligible in both cases, compared to its total concentration, [NaClO_3_]; see below).

Figure 1c exposes the above data for these tests as the relation between the evolutions of the current, *I*, and the normalized charge, *Q*/*Q*^0^. One can see that the shapes of the red and blue lines are close to one another while their markedly different values of the initial concentration of the catalyst merely results in a mutual displacement of these lines along the charge axis.

#### 2.1.2. Chronoamperometry: 6 M H_2_SO_4_; Potential Step to 0.1 V; Series of NaClO_3_ Concentrations

**Figure 2:**
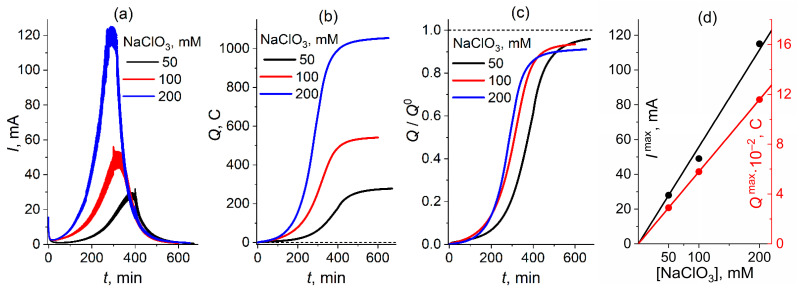
Chronoamperometric data for potential step from OCP to 0.1 V vs. Ag/AgCl (sat. KCl). Series of initial solution compositions: x mM NaClO_3_ + 6 M H_2_SO_4_ where the values of x (from 50 to 200) for each line are indicated in legend. Variation of current, *I* (**a**) or passed reduction charge, *Q* (**b**), or normalized passed reduction charge, *Q/Q*^0^, where *Q*^0^ = 6 *F V* [NaClO_3_] (**c**) as functions of time. Dependences of the maximal current, *I*^max^ (black points) or the maximal charge, *Q*^max^ (at the end of the measurement, red points) on the initial chlorate concentration, [NaClO_3_] (**d**).

The second set of measurements were taken in an analogous way for a lower concentration (6 M) of sulfuric acid while the initial chlorate concentration varies from 50 mM to 200 mM (Figure 2). One can see (Figure 2a) that an increase in the chlorate concentration leads to a higher initial intensity of the catalytic current, to a faster rise in this intensity in time and to an increase in the maximal current (proportionally to the chlorate concentration, with the slope of 0.57 mA/mM, Figure 2d). One can expect that these effects originate from higher concentrations of the chemically generated catalyst and of the chlorate itself.

The corresponding dependences for the passed charge, *Q*, on time, *t*, for these solution compositions are shown in Figure 2b and (in the normalized form) in Figure 2c. Similar to Figure 1b, as the current approaches zero within the long-time range for all chlorate concentrations (Figure 2a), this results in the saturation of the charge value, this upper limit of the charge, *Q*^max^, being approximately proportional to the total reduction charge of the corresponding solution, *Q*^0^ = 6 *F V* [NaClO_3_] (Figure 2c), i.e., to the initial chlorate concentration (with the slope of 5.3 C/mM, Figure 2d). The efficiency of the chlorate-to-chloride transformation varies from 96% for 50 mM (i.e., it is practically identical to that for the 8 M acid solution in Figure 1b) to 91% for 200 mM (Figure 2c).

#### 2.1.3. Chronoamperometry: 8 M H_2_SO_4_; Comparison of Potential Steps to 0.1 V and to 0.7 V

Finally, the dependence on the amplitude of the potential step (up to 0.1 V or up to 0.7 V) has been studied. The other parameters have been identical to those in Figure 1 above, i.e., the initial solution composition has been 50 mM NaClO_3_ + 8 M H_2_SO_4_.

**Figure 3:**
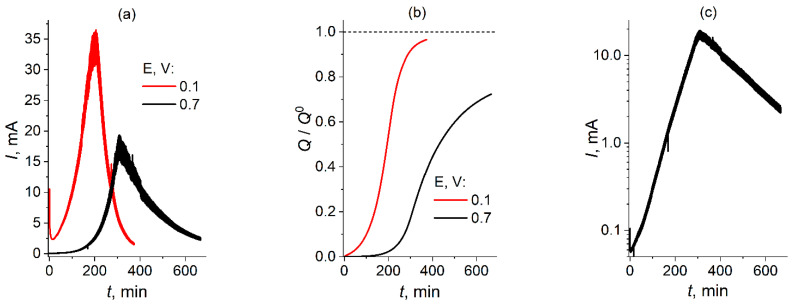
Chronoamperometric data for potential step from OCP to 0.1 V vs. Ag/AgCl (sat. KCl) (red line, see Figure 1) or to 0.7 V vs. Ag/AgCl (sat. KCl) (black line). Solution composition: 50 mM NaClO_3_ + 8 M H_2_SO_4_. Variation of current, *I* (**a**) or normalized passed reduction charge, *Q/Q*^0^, where *Q*^0^ = 6 *F V* [NaClO_3_] (**b**) as functions of time; (**c**) black line of (**a**) (for potential step to 0.7 V vs. Ag/AgCl) in semi-logarithmic coordinates: log *I* vs. *t*.

Figure 3a,b present experimental dependences of the current, *I*, or normalized passed reduction charge, *Q/Q*^0^, on time, *t*, for the potential step up to 0.1 V (red lines, Figure 1a,b) or up to 0.7 V (black lines).

The lines for the current have a similar shape: an initial period of the current growth with an increasing slope up to a narrow maximum, followed by a similarly rapid decrease. Figure 3c demonstrates that for the smaller-amplitude potential step (up to 0.7 V) both the rising and dropping segments of the current–time dependence in Figure 3a (black line) are close to a *purely exponential* form, with a slight deviation within the initial time interval and a very narrow transition range between the segments in the vicinity of the maximum. According to the theoretical analysis for the EC-autocat mechanism [33], such an exponential increase in the current is a *characteristic manifestation of the autocatalytic feature* of the process under study; see also the analysis below.

The charge–time plots in Figure 3b also have a similar shape: an increase in the charge with an increasing rate within the initial time interval, followed by an approach to a maximal value, *Q*_max_. This upper limit is not reached for the smaller-amplitude potential step (up to 0.7 V, black line), maybe because of an insufficiently extended long-time range: see the black line in Figure 3a where the decrease in the current still continues at the end of the measurement time interval.

Even though the general shapes of the dependences in Figure 3a,b are similar for red and black lines, one can see that the amplitude of the potential step strongly affects *quantitative* features of these responses. First, the smaller amplitude leads to a *much slower* increase in the current and, consequently, of the charge. Second, the intensity of the maximal current for the black line diminished markedly, compared to that for the red line. On the contrary, the integral characteristic of the process, i.e., the *maximal charge* passed within the whole duration of the process, changes less significantly: a decrease to about 72% (black line in Figure 3b).

One should emphasize that such an effect of the amplitude of the imposed potential after the step would *not* take place at the transport-limited plateau of the current *if the reaction mechanism does not change*. Thus, one may expect a difference between the mechanisms dependent on the amplitude of the potential step.

In this study, we carried out a *quantitative analysis* of the mechanism of the chlorate-reduction process for a *smaller-amplitude* potential step, up to 0.7 V.

### 2.2. Spectroscopic Studies of Chlorate Reduction Process. Comparison of Current and Chlorine Dioxide Evolutions

Synchronic measurements of the electrochemical and spectroscopic data represent a key tool for establishing a kinetic scheme of the chlorate reduction process. In the preliminary study of this problem [56], it was revealed that a large amount of chlorine dioxide, ClO_2_, was generated in the course of the process. It should be emphasized that the ClO_2_ concentration was varying in time in parallel to the passing current, *by several decimal orders of magnitude*, while the maximal value of this concentration was *comparable with the initial concentration of chlorate*. It was concluded on the basis of these observations that chlorine dioxide represents a principal intermediate of the whole process.

In this study, such a synchronized measurement of the current and UV–visible spectra was carried out in the course of voltammetric experiments for the potential step up to 0.7 V vs. Ag/AgCl (Figure 3). It is these data which have been used below for their interpretation on the basis of a kinetic scheme.

Such a value of the potential after the step was chosen in view of several points:

(1) Standard potential of the one-electron reduction of ClO_2_ to HClO_2_ is above 1.1 V vs. SHE, i.e., above 0.9 V vs. Ag/AgCl (sat. KCl), within this pH range [75], while this process takes place in a quasi-reversible manner at platinum electrode [65,66]; it means that the chosen potential, 0.7 V vs. Ag/AgCl (sat. KCl), is sufficient for passage of the ClO_2_ reduction under the transport-limited conditions;
(2) Subsequent transformation of the product of the ClO_2_ electroreduction, HClO_2_, via the chemical (disproportionation, see below) mechanism, was already extensively studied, see, e.g., [62,63,64,65,66,67,68,69,70,71,72,73]; this information is used below for the formulation of the kinetic scheme of the *chlorate reduction* process; a greater amplitude of the potential step (up to 0.1 V vs. Ag/AgCl) can lead to *extra electrochemical stages* due to reaction(s) of HClO_2_ or other Cl-containing components of lower oxidation degrees, thus perturbating the *chemical* scheme of the HClO_2_ transformation;
(3) This study of the chlorate reduction inside an electrochemical cell has been performed in order to get extra information concerning the same process taking place inside the H_2_-ClO_3_^−^ power source where the usual potential of the positive electrode (vs. Ag/AgCl) in the course of the current generation belongs frequently to the range in the vicinity of about 0.5 V, very far from its value of 0.1 V [57].

**Figure 4:**
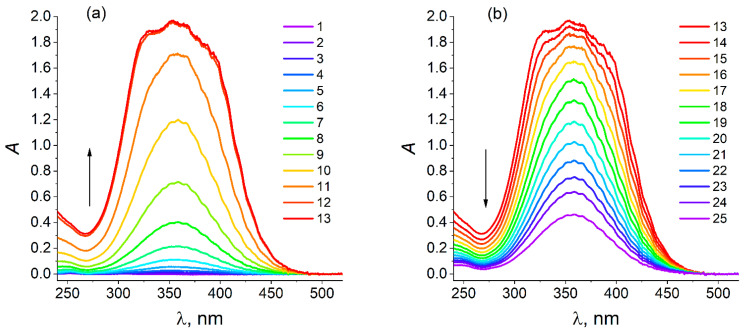
Evolution of the registered UV–visible spectrum of the electrolyte solution in the course of the chronoamperometric measurement for potential step to 0.7 V (current evolution: black line in Figure 3a): time range of increasing current and absorption, lines 1 to 13 (**a**); time range of decreasing current and absorption, lines 13 to 25 (**b**).

A set of spectra of the solution in the course of the chronoamperometric study for the potential step up to 0.7 V (black line for the current in Figure 3a) is given in Figure 4. Within the initial time range the absorption within a broad band around 360 nm (related to solute ClO_2_) rapidly increases (Figure 4a), up to the absorption saturation within the medium range of the band. After the moment when the current passes through its maximum (black line in Figure 3a), the intensity of the solution absorption decreases (Figure 4b).

In view of strongly acidic conditions, the solution in the course of the chlorate electrolysis can contain its corresponding reduction products: ClO_2_, HClO_2_, HClO, Cl_2_, Cl_3_^−^ and Cl^−^ (a further discussion is given in subsequent sections). For the analysis of the solution composition, the wavelength interval of its spectrum (Figure 4) between 358 nm and 440 nm was used since it is well known (see, e.g., [51,67,73,76]) that within this range, *one may disregard* the contributions to the spectrum from all Cl-containing components, *except for chlorine dioxide*, ClO_2_, both because of its very broad absorption band around 358 nm and its large extinction coefficient, which greatly exceeds those of other solute components.

A special treatment of these spectral data has been proposed in order to determine the ClO_2_ concentration for each of the solution spectra in Figure 4. This procedure is described in Section S2.

**Figure 5:**
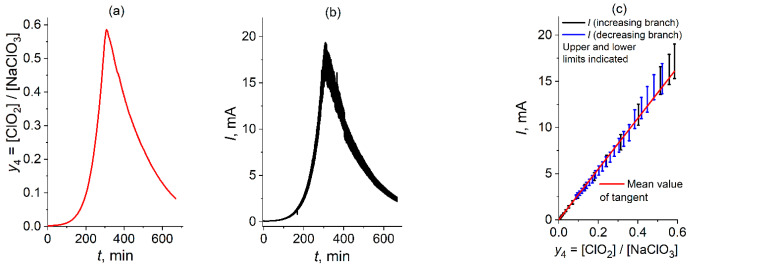

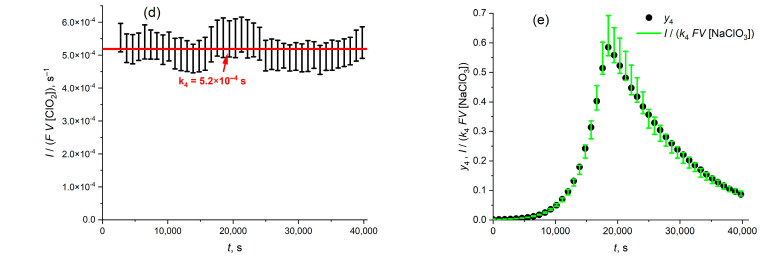
Evolutions of dimensionless ClO_2_ concentration, *y*_4_ = [ClO_2_]/[NaClO_3_] (**a**) and of current, *I* (copy of black line in Figure 3a) (**b**) in the course of chronoamperometric study for the potential step from OCP to 0.7 V vs. Ag/AgCl (sat. KCl). Presentation of data in (**a**,**b**) for a discrete set of time moments in the coordinates: current, *I*, vs. dimensionless ClO_2_ concentration, *y*_4_ = [ClO_2_]/[NaClO_3_], for both their increasing and decreasing branches (**c**); ratio of current to ClO_2_ concentration, *I*/(*F V* [ClO_2_]), vs. time, *t* (**d**); dependences: *y*_4_ and *I*/(*k*_4_ *F V* [NaClO_3_]), vs. time, *t* (**e**), where *F* is the Faraday constant, *V* is the solution volume. In (**c**–**e**) vertical lines for current indicate its upper and lower limits for the corresponding time moments, compare with the widened line for current in (**b**), due to its rapid fluctuations (see caption to Figure 1 and Section S1 “Materials and Methods” for more details). Solution composition: 50 mM NaClO_3_ + 8 M H_2_SO_4_.

Thus, the evolution of the chlorine dioxide concentration, [ClO_2_], in the course of the chronoamperometric experiment is shown in Figure 5a for its dimensionless value, i.e., the ratio of this concentration to the initial concentration of chlorate prior to the experiment: *y*_4_ = [ClO_2_]/[NaClO_3_], where [NaClO_3_] = 50 mM. The corresponding chronoamperometric plot, *I* vs. *t,* is given in Figure 5b for the sake of comparison.

One can see an obvious analogy between the shapes of the plots in Figure 5a,b. A rapid (exponential-like, see Figure 3c for the current) increase from very low initial values, with subsequent passage of a narrow maximum, followed by a rapid (again exponential-like, see Figure 3c for the current) decrease. This shape may be considered *a direct indication* in favor of the *autocatalytic* character of the whole process, which represents another example of the *autocatalytic redox-mediator cycle* mechanism (EC-autocat, earlier notation: EC” [13,32,33]).

Moreover, a thorough analysis shows that the maxima of both plots are located at *the same time moment* (about 18,560 s, i.e., 30 min). This coincidence of the maximum’s moments for the ClO_2_ concentration and the current is a *well-reproducible* observation; see, e.g., the same result found for the chlorate electroreduction process *under quite different conditions* [56].

A base for the *quantitative* analysis is given in Figure 5c for the relation between the experimental values of the current, *I*, and the dimensionless ClO_2_ concentration (*y*_4_) *at the same time moment*, *t*, *for a broad set of time moments*. Each point is given with indication of the maximal and minimal values of the current for this moment, which reflect the widened interval for the current values in Figure 5b; see the description of the origin of these current fluctuations in the caption of Figure 1 and Section S1 “Materials and Methods”.

One should note first of all that the points for the *increasing* and *decreasing* branches in Figure 5a,b are *overlapping* in Figure 5c (black and blue points, respectively). Moreover, all these points are located *near a straight (red) line passing through the origin*. It means that the experimental values of the current and of the ClO_2_ concentration at any time are *proportional between one another*, *the proportionality constant being the same within the whole time range*, including the increasing and decreasing branches in Figure 5a,b.

This very important result directly originating from the above experimental data is *well reproducible* in our other experiments; in particular, it was already observed in the preliminary study [56] for quite different values of parameters, in particular, the initial concentrations, [NaClO_3_] and [ClO_2_]^0^.

Figure 5d presents the same observation in different coordinates: as a plot for the *ratio of the current*, *I*, *to the chlorine dioxide concentration*, [ClO_2_], *at the same time moment*, *t* (in the form: *I*/(*F V* [ClO_2_]), *F* is the Faraday constant, *V* is the solution volume) as a function of time. In conformity with Figure 5c, the data for the ratio in Figure 5d are *close to a constant value within the whole range of the electrolysis time*, *t*, *without any regular variation in time*. Moreover, the *average* value of this ratio shown in Figure 5d by a *horizontal red line lies between the upper and lower limits of the ratio for all time moments*.

It means that this ratio averaged over the fluctuations is *constant* within the whole time range.

(10) $$I_{aver}=k_{4}F\;V\left[{\mathrm{ClO}}_{2}\right]\;\mathrm{where}\;k_{4}=5.2\;10^{-4}s$$

where *I*_aver_ is the current averaged over its fluctuations.

Finally, Figure 5e superimposes experimental data for the dimensionless ClO_2_ concentration, *y*_4_ = [ClO_2_]/[NaClO_3_], and for the normalized current, *I*/(*k*_4_ *F V* [NaClO_3_]), as functions of time, *t*, *for the chosen set of time moments*. In conformity with Equation (10) *both lines coincide if the dispersion of the current is taken into account*.

These experimental observations (Figure 5c–e) and their consequence, relation (10), will be of *great importance for the choice of the mechanism of the whole chlorate reduction process*. Namely, it implies that the whole kinetic scheme of the process includes *only one electrochemical step* (which determines the passing current under conditions of the transport-limited regime), *where chlorine dioxide acts as a reactant*.

It is known that chlorine dioxide may be *reduced reversibly* via *one-electron* process [74] under acidic conditions, see scheme (7), so that the parameter in Equation (10), *k*_4_, plays the role of an *effective reaction constant of electrochemical step* (7) *within the diffusion-limited current regime* while the product, *k*_4_ [ClO_2_], is equal to the rate of the concentration change: – d[ClO_2_]/d*t* due to reaction (7), *v*_4_:

(11) $$v_{4}=\;k_{4}{\;[\mathrm{ClO}}_{2}]$$

Here and below, the subindices of the rates and rate constants of the stages of the global process mean *the highest oxidation degree of a reactant*, e.g., subindex “4” implies to the oxidation degree “4” for the ClO_2_ electroreduction.

One should keep in mind that in acidic solutions, the dissociation of chlorous acid is practically suppressed in view of the value of its acidic-dissociation constant, pK = 1.72 [60], while the standard potential of reaction (7) for this acidic pH range is given by the relation *E*^0^ [V] = 1.143 – 0.059 pH on the basis of data in [75]. According to [53,54], if a high-acidity chlorate solution is kept *in darkness* after its preparation, no further chlorate decomposition takes place, compared to that in the course of the solution preparation.

### 2.3. Kinetic Schemes of Chlorate Reduction Process

#### 2.3.1. Comproportionation Reaction Between Cl(V) and Cl(III) Compounds

Comparison of synchronic experimental data for the chronoamperometric current and the ClO_2_ concentrations under various conditions (see, e.g., above and those in [56]) confirms the absence of a direct electroactivity of the chlorate ion (even in the presence of a high acid concentration), at least on non-modified carbonaceous electrodes. Namely, the current passing immediately after formation of the mixed chlorate-acid solution is very low and its value is irreproducible, being dependent on details of the mixing procedure. Current increase during the subsequent time interval occurs in parallel with the rise of the ClO_2_ concentration, see Equation (10) and Figure 5c. It means that starting conditions for the further chlorate reduction process are determined by a *very slight* chemical decomposition of chlorate ions, with formation of various Cl-containing species, including chlorine dioxide. Variation of their concentrations may affect markedly the subsequent evolution of the global chlorate reduction process (see, e.g., Figure 1a), first of all via a change of the duration of the induction period. If needed (e.g., for the use of the chlorate process in power sources), this duration may be strongly diminished by a special choice of the electrochemical regime [58].

In view of the non-electroactivity of chlorate anions at the electrode, the kinetic scheme of the global process has to include at least one *chemical* step with participation of ClO_3_^−^.

One of the candidates is its reaction with the *chloride* anion, which was studied experimentally in numerous publications [39,40,41,42,43,44,45,46]. According to a discussion in the Introduction section, the reaction between chlorate and chloride can follow two parallel routes, Equations (5) and (6), the transformation rate being very slow [39]. Additionally, the principal route, Equation (6), leads to generation of *two products*, ClO_2_ and Cl_2_, *both of them being electroactive at electrochemical conditions under study*. At the same time, experimental results presented in Figure 5 *prove unanimously* that *ClO_2_ is the only electroactive species within the whole time range of the electrochemical experiment*, in direct contradiction to the information available for the chlorate–chloride reaction. On this basis, one should expect that *the generation of a rapidly increasing amount of ClO_2_* (Figure 5a) is a consequence of *another redox-mediator cycle*.

Therefore, it looks more probable that this accumulation of ClO_2_ is primarily due to a *comproportionation reaction (8) between chlorate anions with the product of electrochemical step (7)*, HClO_2_, which *might* give chlorine dioxide. It is evident from the schemes of these electrochemical and chemical steps that they form an *autocatalytic redox cycle*, in conformity with the shape of plots in Figure 5a,b.

It should be noted that the kinetics of this chemical step was mostly studied in neutral, alkaline or weakly acidic solutions (see, e.g., [71]) where this reaction proceeds in the *opposite* direction, i.e., as a *chlorine dioxide disproportionation*. Its scheme may be written down in two equivalent forms:

(12) $${2\;\mathrm{ClO}}_{2}{+\;H}_{2}O\;=\;{{\mathrm{ClO}}_{3}}^{-}+\;{{\mathrm{ClO}}_{2}}^{-}{+\;2\;H}^{+}{\;\mathrm{or}\;2\;\mathrm{ClO}}_{2}{+\;2\;\mathrm{OH}}^{-}=\;{{\mathrm{ClO}}_{3}}^{-}+\;{{\mathrm{ClO}}_{2}}^{-}{+\;H}_{2}O$$

The value of its equilibrium constant, *K*_disp(base)_, may be found from the standard potentials for transformations of the Cl-containing components in Equation (12), see, e.g., [75]:

(13) $$\log K_{\mathrm{disp}(\mathrm{base})}=\log\left(\frac{\left[{\mathrm{ClO}}_{3}^{-}][{\mathrm{ClO}}_{2}^{-}\right]}{{\left[{\mathrm{ClO}}_{2}\right]}^{2}}\right)=-2.904+2\mathrm{pH}$$

which predicts its high values within the whole range of neutral and alkaline media. As for its rate, it becomes significant only at high pH values, while the measurements give very low values for the rate constant in neutral and weakly acidic solutions [71].

The situation changes within the range of acidic solutions. First of all, the acidic dissociation of HClO_2_ is suppressed since the reported value of the pK_a_ value of this process:

(14) $${\mathrm{HClO}}_{2}{=\;\mathrm{ClO}}_{2}^{-}{+\;H}^{+},\;\;pK_{a}=-\log K_{a}=\log[{\mathrm{HClO}}_{2}]-\log[{\mathrm{ClO}}_{2}^{-}]+\mathrm{pH}$$

is 1.72 [60] (1.96 in Ref. [61]). As a result, for a strong-acid concentration within the molar range, one should rewrite equilibrium (12) as the *comproportionation* reaction between ClO_3_^−^ and HClO_2_; see scheme (8). The value of its equilibrium constant, *K*_disp(acid)_, may also be found from the standard potentials for transformations of the Cl-containing components in Equation (8); see, e.g., [75]:

(15) $$\log K_{\mathrm{disp}(\mathrm{acid})}=\log\left(\frac{{\left[{\mathrm{ClO}}_{2}\right]}^{2}}{\left[{{\mathrm{ClO}}_{3}}^{-}\right]\left[{\mathrm{HClO}}_{2}\right]}\right)=1.184-\mathrm{pH}$$

Thus, for *negative* pH values, equilibrium (8) is strongly shifted *to the right*, i.e., the generation of chlorine dioxide is *energetically favorable*.

Unfortunately, the direct kinetic information of the *rate* of this chemical step at high acid concentrations is not available. It may be expected that in our experimental conditions (8 M H_2_SO_4_ solution), the rate-determining step consists of the reaction between the neutral forms of both acids: O_2_Cl-OH + H-OClO, with simultaneous H-atom transfer from HClO_2_ to give O_2_Cl-OH_2_ and bond-breaking between Cl and OH_2_ in chlorate. Then, the rate of step (8), *v*_5_, should be proportional to the product of the reactants’ concentrations:

(16) $$v_{5}=k_{5}[{{\mathrm{ClO}}_{3}}^{-}]\;[{\mathrm{HClO}}_{2}]$$

Combination of the electrochemical reduction of ClO_2_, Equation (7), with this chemical step, Equation (8), represents a *redox-mediator cycle* that is able to transform progressively the principal non-electroactive component, ClO_3_^−^ anion, into Cl-containing species of lower oxidation degrees, +4 and +3. It obviously has an *autocatalytic* feature since passage of this cycle leads to a *gradual accumulation of the components of the catalytic redox couple*: ClO_2_/HClO_2_, i.e., it corresponds to the EC-autocat (EC”) mechanism [32].

An analogous autocatalytic redox-mediator cycle was proposed in [52] in order to transform a highly acidic (at least 7 N H_2_SO_4_) chlorate solution into chlorine dioxide. A similar scheme of the chlorate reduction process as a route for ClO_2_ production via the chlorate electrolysis was also postulated in [53,54]. However, the postulated cycle included ClO_2_^−^ *anion* in both the electrochemical and comproportionation steps, instead of the *acid*, HClO_2_, while this ion does *not exist* at such high acidities.

#### 2.3.2. Disproportionation Reactions of Cl(III) Compounds [62,63,64,65,66,67,68,69,70,71,72,73]

The above kinetic scheme of the chlorate reduction process consisting of steps (7) and (8) would result finally in HClO_2_, i.e., to the transfer of *two electrons per one chlorate anion*, so that the ratio of the passed charge to its value for the chlorate-to-chloride transformation, *Q*/*Q*^0^, *should not exceed 33%*. This contradicts the experimental data in Figure 3b (black line) that suggest *this ratio reaches a much higher value,* 72%, i.e., the transfer of *more than four electrons*. This means that HClO_2_ is an *intermediate* product of the process, rather than a *final* one. This result, which is crucially important for energetic applications of the chlorate reduction process, has also been achieved in our hydrogen/chlorate power sources [57,58].

This situation is a consequence of the *instability* of HClO_2_ with respect to the *disproportionation* reaction, which is able to generate *the chloride anion*.

According to the above literature information, this step can proceed via *several* different *chemical schemes*:

##### Scheme A

If an *acidic* solution of HClO_2_ is in contact with the surface of a solid platinum (either with an electrically isolated Pt material or with a Pt electrode), this substance is transformed into ClO_2_ and Cl^−^ [65,66] via a set of elementary chemical steps, resulting in the global reaction:

(17) $${5\;\mathrm{HClO}}_{2}{=\;4\;\mathrm{ClO}}_{2}{+\;\mathrm{Cl}}^{-}{+\;H}^{+}{+\;2\;H}_{2}O$$

Kinetically, the whole process is limited by a step catalyzed heterogeneously by the Pt surface so that the rate of process (13) *v*_3A_ is proportional to the HClO_2_ concentration:

(18) $$\frac{d[{\mathrm{Cl}}^{-}]}{dt}=v_{3A}=k_{3A}[{\mathrm{HClO}}_{2}]$$

where the rate constant, *k*_3A_, is proportional to the activity of protons.

All alternative schemes of the HClO_2_ disproportionation below (B, C, BC and KG) [62,63,64,67,68,69,70,71,73] are based on a set of chemical steps *inside the solution phase* initially containing HClO_2_ (or chlorite in neutral or alkaline medium); in some cases, other chlorine-containing components were added, e.g., Cl^−^ or ClO_3_^−^ anions. The main products of this reaction were ClO_2_, Cl^−^ and ClO_3_^−^ species.

##### Scheme B

In particular, in acidic medium *in the absence* (or at *sufficiently low* concentrations) of chloride anions in solution, the HClO_2_ disproportionation follows a kinetic scheme [62,67] *different* from scheme A:

2 HClO_2_ = ClO_3_^−^ + HClO + H^+^ (rate-determining step),

HClO + 2 HClO_2_ = 2 ClO_2_ + Cl^−^ + H^+^ + H_2_O (set of rapid steps)

which gives Equation (19) for the total disproportionation process:

(19) $${4\;\mathrm{HClO}}_{2}{=\;2\;\mathrm{ClO}}_{2}+\;{{\mathrm{ClO}}_{3}}^{-}{+\;\mathrm{Cl}}^{-}{+\;2\;H}^{+}{+\;H}_{2}O$$

Its rate, *v*_3B_, is determined by the primary step so that

(20) $$\frac{d[{\mathrm{Cl}}^{-}]}{dt}=v_{3B}=k_{3B}{\left[{\mathrm{HClO}}_{2}\right]}^{2}$$

##### Scheme C

The expressions of scheme B change cardinally in the presence of a *sufficiently high* concentration of chloride anions [67] where the primary step is different:

HClO_2_ + Cl^−^ + H^+^ = 2 HClO (rate determining step),

HClO + 2 HClO_2_ = 2 ClO_2_ + Cl^−^ + H^+^ + H_2_O (set of rapid steps)

Which gives this time Equation (17) for the total disproportionation process *identical to that of scheme A*, but its rate is given to scheme C by Equation (21), determined by the primary step of the process:

(21) $$\frac{d[{\mathrm{Cl}}^{-}]}{dt}=v_{3C}=k_{3C}[{\mathrm{HClO}}_{2}][{\mathrm{Cl}}^{-}]$$

##### Scheme BC

This scheme represents a combination of schemes B and C for the case where the solution of HClO_2_ also contains Cl^−^, their concentrations being comparable with one another. Then, one may expect that the HClO_2_ disproportionation proceeds inside the solution phase via *two parallel mechanisms*, which start from the HClO_2_ reactions either with another HClO_2_ molecule or with the Cl^−^ anion, respectively, where these primary steps are *rate-determining*. If these processes pass *in an independent way*, then one may use for them Equations (17) and (19), respectively, so that the HClO_2_ disproportionation rate will be given by the sum of their contributions in Equations (20) and (21), thus resulting in expression (22):

(22) $$\frac{d[{\mathrm{Cl}}^{-}]}{dt}=v_{\text{3BC}}≅k_{3B}{\left[{\mathrm{HClO}}_{2}\right]}^{2}+k_{3C}[{\mathrm{HClO}}_{2}{][\mathrm{Cl}}^{-}]$$

As is indicated below, scheme BC may also be derived as *an approximation* of the expression for the HClO_2_ disproportionation rate for scheme KG.

##### Scheme KG

The most general expression for the rate of the HClO_2_ disproportionation (denoted as scheme KG below) was substantiated *empirically* by Kieffer and Gordon [73]. It is based on their experimental data for the evolution of the concentrations of all Cl-containing components, both in the absence and in the presence of Cl^−^ ions, for various initial concentrations of both components in solution as well as for several concentrations of the background electrolyte, HClO_4_.

It has the following form for the rate of the HClO_2_ consumption due to the its disproportionation:

(23) $$-\frac{d[{\mathrm{HClO}}_{2}]}{dt}=k_{1}{\left[{\mathrm{HClO}}_{2}\right]}^{2}+\frac{k_{2}[{\mathrm{HClO}}_{2}{][\mathrm{Cl}}^{-}{]}^{2}}{K+[{\mathrm{Cl}}^{-}]}$$

where two parameters are independent of pH, *k*_1_ = 1.17 × 10^−2^ M^−1^ s^−1^, *K* = 1.2 mM, while *k*_2_ depends on pH: *k*_2_ is equal to 1.57 × 10^−2^ M^−1^ s^−1^ for [H^+^] = 1.2 M and 3.00 × 10^−2^ M^−1^ s^−1^ for [H^+^] = 2 M [73].

In view of kinetic schemes (19) and (17) related to the first and to the second contributions into Equation (23), respectively, the expression for the rate of the *Cl^−^ generation from HClO_2_* takes the form:

(24) $$\frac{d{[\mathrm{Cl}}^{-}]}{dt}=v_{\text{3KG}}=k_{3B}{\left[{\mathrm{HClO}}_{2}\right]}^{2}+\frac{k_{3C}[{\mathrm{HClO}}_{2}{][\mathrm{Cl}}^{-}{]}^{2}}{K+[{\mathrm{Cl}}^{-}]}$$

where *k*_3B_ = *k*_1_/4 = 0.29 × 10^−2^ M^−1^ s^−1^, *k*_3C_ = *k*_2_/5 = 0.31 × 10^−2^ M^−1^ s^−1^.

This scheme of the HClO_2_ disproportionation process assumes its passage *inside the solution phase* via *two parallel independent mechanisms* described above as schemes B and C, i.e., starting from the HClO_2_ reactions either with another HClO_2_ molecule or with Cl^−^ anion, respectively.

The rate of the former is determined by its primary (irreversible) step so that expression (20) is again valid.

On the contrary, the rate of the latter mechanism is written down by an empirical expression: *v*_3C_ = *k*_3C_ [HClO_2_] [Cl^−^]^2^/(*K* + [Cl^−^]); see Equation (24). According to Equations (28) and (29) in [73], this rate is determined *by two primary steps*:

(25) $${\mathrm{HClO}}_{2}{+\;\mathrm{Cl}}^{-}=\;\left[{\mathrm{HCl}}_{2}{O_{2}}^{-}\right],\;\left[{\mathrm{HCl}}_{2}{O_{2}}^{-}\right]{\;+\;\mathrm{Cl}}^{-}=\;\mathrm{products}\;\left(\mathrm{series}\;\mathrm{of}\;\mathrm{steps}\right)$$

Step 1 is assumed to be *in equilibrium* so that the concentration of its product is given by the relation [HCl_2_O_2_^−^] ≅ *K*_eq_ [HClO_2_] [Cl^−^], i.e., [HCl_2_O_2_^−^] ≅ *K*_eq_ [HClO_2_]^0^ [Cl^−^]/(1 + *K*_eq_ [Cl^−^]), where [HClO_2_]^0^ is the HClO_2_ concentration *prior to* the Cl^−^ addition. Step 2 is considered *irreversible* (k_2_ being its effective rate constant) so that one has an expression for the reaction rate:

(26) $$v_{3C}=\frac{d[{\mathrm{Cl}}^{-}]}{dt}=k_{2}[{\mathrm{HCl}}_{2}O_{2}^{-}{][\mathrm{Cl}}^{-}]\approx \frac{k_{2}{\left[{\mathrm{HClO}}_{2}\right]}_{0}{{[\mathrm{Cl}}^{-}]}^{2}}{K_{\mathrm{eq}}^{-1}+[{\mathrm{Cl}}^{-}]}$$

which is identical to the second contribution into expression (24), where the pH-independent parameter, *K*, is equal to 1/*K*_eq_, while the pH-dependent one, *k*_2_, is equal to the *k*_3C_ parameter.

This derivation of Equation (26) for *v*_3C_ assumes *implicitly* that the Cl^−^ addition into the HClO_2_ solution *changes rapidly* (compared to the temporal scale of the global process, Figure 5) its concentration prior to chloride addition, [HClO_2_]^0^, into the *mixture* of *a lower concentration*, [HClO_2_], and that of the product, [HCl_2_O_2_^−^]. On the other hand, this change in the HClO_2_ concentration due to the Cl^−^ addition is *implicitly disregarded* in the expression of the B contribution: *v*_3B_ = *k*_3B_ [HClO_2_]^2^, where the concentration, [HClO_2_], is assumed *to remain unchanged, despite the Cl^−^ addition into solution*. However, within the framework of the above model, its value in the expression for *v*_3B_ should be replaced by its expression: [HClO_2_]^0^/(1 + *K*_eq_ [Cl^−^]) *which depends on the Cl^−^ concentration*, i.e., its rate, *v*_3B_, *depends on the Cl^−^ concentration*. Therefore, the presence of Cl^−^ ions in solution affects *both contributions to the rate of the HClO_2_ disproportionation,* i.e., Equation (24) is *not valid within the framework of these kinetic assumptions* of [73].

We propose a different derivation of Equation (24) on the basis of other kinetic assumptions, which, in particular, justify the postulate on two independent mechanisms of the HClO_2_ transformation.

Let us assume that the rate of the C mechanism is determined by kinetics of two primary steps that are slightly different from those in [73] in order to take into account pH-dependences of the parameters:

(27) $${\mathrm{HClO}}_{2}{+\;\mathrm{Cl}}^{-}{+\;H}^{+}=\;\left[H_{2}{\mathrm{Cl}}_{2}O_{2}\right],\;\left[H_{2}{\mathrm{Cl}}_{2}O_{2}\right]{\;+\;\mathrm{Cl}}^{-}=\;\mathrm{products}\;\left(\mathrm{series}\;\mathrm{of}\;\mathrm{steps}\right)$$

where the rates of the steps 1 and 2, v_1_ and v_2_, are given by the expressions:

(28) $$v_{1}=\;k_{f}\left[{\mathrm{HClO}}_{2}\right]\;\left[{\mathrm{Cl}}^{-}\right]\;-\;\;k_{b}\left[H_{2}{\mathrm{Cl}}_{2}O_{2}\right],\;\;v_{2}=\;k’\;\left[H_{2}{\mathrm{Cl}}_{2}O_{2}\right]\;\left[{\mathrm{Cl}}^{-}\right]\;$$

Here, *k*_f_ and *k*_b_ are the forward and the backward constants of step 1, and *k*’ is the effective rate constant of the second (irreversible) step. Within the framework of the approximation of steady-state concentrations (applied to the intermediate complex, [H_2_Cl_2_O_2_]) one has for the total disproportionation reaction rate via this route, *v*_3C_:

(29) $$\begin{array}{l} v_{3C}≅v_{1}≅v_{2},\;i.e.\;k_{f}[{\mathrm{HClO}}_{2}{][\mathrm{Cl}}^{-}]≅(k_{b}+k^{\prime }[{\mathrm{Cl}}^{-}{])[H}_{2}{\mathrm{Cl}}_{2}O_{2}],\;\;\mathrm{and}\\ v_{3C}≅k^{\prime }[H_{2}{\mathrm{Cl}}_{2}O_{2}{][\mathrm{Cl}}^{-}]≅\frac{k^{\prime }k_{f}[{\mathrm{HClO}}_{2}{][\mathrm{Cl}}^{-}{]}^{2}}{k_{b}+k^{\prime }[{\mathrm{Cl}}^{-}]}=\frac{k_{f}[{\mathrm{HClO}}_{2}{][\mathrm{Cl}}^{-}{]}^{2}}{k_{b}/k^{\prime }+[{\mathrm{Cl}}^{-}]} \end{array}$$

This expression for the rate of this mechanism (denoted C in this paper) is again identical to Formula (24) if

(30) $$K=\frac{k_{b}}{k’},\;k_{3C}=k_{f}$$

One should note that, according to our interpretation of this formula, the *K* parameter is *not* the equilibrium constant of step 1, *k*_b_/*k*_f_, but it represents a ratio of the rate constants of steps 1 and 2, *k*_b_/*k*’, while another parameter of Equation (24), *k*_3C_, is the rate constant of step 1 rather than that of step 2, as it was in the derivation in [73]; see above.

According to Equation (30) the pH-independent *K* parameter is equal to the ratio of two different ways of the complex, [H_2_Cl_2_O_2_], decomposition while another parameter, *k*_3C_, increases parallel to the acid concentration, in conformity with kinetic scheme (27) rather than with scheme (25).

Another advantage of kinetic scheme (27) is the absence of step 2 of scheme (25) where *both reactants are negatively charged* so that they have to overcome a *repulsive barrier*.

Within the time range where the chlorous acid concentration, [HClO_2_], is much larger than 1 mM, the second term in Equation (24) gives a marked contribution comparable with the first one only if the chloride concentration is sufficiently high: [Cl^−^] ≫ *K* ~ 1 mM. Then, the constant term, *K*, in the denominator of expression (24), *K* + [Cl^−^], is small compared to the second one, [Cl^−^]. If one *neglects the former term*, general expression (24) is reduced to that for scheme BC, Equation (22).

For a sufficiently high initial concentration of chlorate ion, ClO_3_^−^ (e.g., for *c*_5_^0^ = [ClO_3_^−^] ≅ 50 mM, i.e., much larger than the *K* value) it *seems* that the whole time range may be separated into two intervals. Within *the shorter time interval,* the second contribution into Equation (24) may be *neglected* because of a very low Cl^−^ concentration. As for *the more extended time range,* the Cl^−^ concentration, [Cl^−^], is already much larger than the constant, *K*, in Equation (24). Therefore, it *seems* from this reasoning that the predictions of schemes BC and KG, Equations (22) and (24), respectively, should be *close to one another*.

It will be shown below that this conclusion is *valid* concerning the general features of the predicted theoretical results. On the other hand, even though the correction due to the extra term, *K*, in the denominator of Equation (24) is *relatively small,* its effect is of importance in the course of the fitting of the theoretical results to experimental data.

One should note that the rate constant in the first term is known from the above data: *k*_3B_ = 0.29 × 10^−2^ M^−1^ s^−1^, while that in the second term, *k*_3C_, depends on pH so that for the 8 M H_2_SO_4_, it should be *much larger than its value for the 2 M HClO_4_*, i.e., 0.60 × 10^−2^ M^−1^ s^−1^.

### 2.4. Global Mechanism of Chlorate-Reduction Process

This mechanism includes mutual transformations of the reagent, ClO_3_^−^, two intermediate Cl-containing species, ClO_2_ and HClO_2_, as well as the final product, Cl^−^, via three stages.

(1) Electrochemical step at the electrode surface:

(31) $${\mathrm{ClO}}_{2}{+H}^{+}{+e}^{-}{=\mathrm{HClO}}_{2}$$

its rate, *v*_4_, being given by relation (11).

(2) Comproportionation reaction between chlorate anion and chlorous acid, with generation of chlorine dioxide:

(32) $${\mathrm{HClO}}_{2}+{{\mathrm{ClO}}_{3}}^{-}{+H}^{+}{=2\;\mathrm{ClO}}_{2}{+H}_{2}O$$

its rate, *v*_5_, being given by relation (16).

(3) Disproportionation reaction of chlorous acid, which can pass via two parallel schemes, with generation of chlorine dioxide, chlorate anion and chloride anion:

(33) $${4\;\mathrm{HClO}}_{2}{=2\;\mathrm{ClO}}_{2}+{{\mathrm{ClO}}_{3}}^{-}{+\mathrm{Cl}}^{-}{+2\;H}^{+}{+H}_{2}O\;\mathrm{and}\;{5\;\mathrm{HClO}}_{2}{=4\;\mathrm{ClO}}_{2}{+\mathrm{Cl}}^{-}{+H}^{+}{+2\;H}_{2}O$$

where the rate of this reaction, *v*_3_, depends on the choice among five schemes: A, B, C, BC and KG discussed in Section 2.3.2 above.

### 2.5. Set of Kinetic Equations for Non-Stationary Concentrations of Solute Cl-Containing Components

Temporal variation in the concentrations of all Cl-containing components: ClO_3_^−^, ClO_2_, HClO_2_ and Cl^−^, is determined by superposition of the contributions due to the three stages listed in Section 2.4 by relations (31), (32) and (33) (proportional to *v*_4_, or *v*_5_, or *v*_3_, respectively).

In view of the existence of five different schemes of the HClO_2_ disproportionation: A, B, C, BC and KG (see above), the set of kinetic equations for the Cl-containing solution component takes the corresponding forms:

Scheme A:

(34) $$\begin{array}{l} \frac{d[{\mathrm{ClO}}_{3}^{-}]}{dt}=-k_{5}[{\mathrm{ClO}}_{3}^{-}{][\mathrm{HClO}}_{2}];\;\;\;\frac{d[{\mathrm{ClO}}_{2}]}{dt}=k_{5}[{\mathrm{ClO}}_{3}^{-}{][\mathrm{HClO}}_{2}]-k_{4}[{\mathrm{ClO}}_{2}]+4k_{3A}[{\mathrm{HClO}}_{2}];\\ \frac{d[{\mathrm{HClO}}_{2}]}{dt}=-k_{5}[{\mathrm{ClO}}_{3}^{-}{][\mathrm{HClO}}_{2}]+k_{4}[{\mathrm{ClO}}_{2}]-5k_{3A}[{\mathrm{HClO}}_{2}];\;\;\;\frac{d[{\mathrm{Cl}}^{-}]}{dt}=k_{3A}[{\mathrm{HClO}}_{2}] \end{array}$$

Scheme B:

(35) $$\begin{array}{l} \frac{d[{\mathrm{ClO}}_{3}^{-}]}{dt}=-k_{5}[{\mathrm{ClO}}_{3}^{-}{][\mathrm{HClO}}_{2}]+k_{3B}{\left[{\mathrm{HClO}}_{2}\right]}^{2};\frac{d[{\mathrm{ClO}}_{2}]}{dt}=2k_{5}[{\mathrm{ClO}}_{3}^{-}{][\mathrm{HClO}}_{2}]-k_{4}[{\mathrm{ClO}}_{2}]+2k_{3B}{\left[{\mathrm{HClO}}_{2}\right]}^{2};\\ \frac{d[{\mathrm{HClO}}_{2}]}{dt}=-k_{5}[{\mathrm{ClO}}_{3}^{-}{][\mathrm{HClO}}_{2}]+k_{4}[{\mathrm{ClO}}_{2}]-4k_{3B}{\left[{\mathrm{HClO}}_{2}\right]}^{2};\frac{d[{\mathrm{Cl}}^{-}]}{dt}=k_{3B}{\left[{\mathrm{HClO}}_{2}\right]}^{2} \end{array}$$

Scheme C:

(36) $$\begin{array}{l} \frac{d[{\mathrm{ClO}}_{3}^{-}]}{dt}=-k_{5}[{\mathrm{ClO}}_{3}^{-}{][\mathrm{HClO}}_{2}];\frac{d[{\mathrm{ClO}}_{2}]}{dt}=2k_{5}[{\mathrm{ClO}}_{3}^{-}{][\mathrm{HClO}}_{2}]-k_{4}[{\mathrm{ClO}}_{2}]+4k_{3C}[{\mathrm{HClO}}_{2}{][\mathrm{Cl}}^{-}];\\ \frac{d[{\mathrm{HClO}}_{2}]}{dt}=-k_{5}[{\mathrm{ClO}}_{3}^{-}{][\mathrm{HClO}}_{2}]+k_{4}[{\mathrm{ClO}}_{2}]-5k_{3C}[{\mathrm{HClO}}_{2}{][\mathrm{Cl}}^{-}];\frac{d[{\mathrm{Cl}}^{-}]}{dt}=k_{3C}[{\mathrm{HClO}}_{2}{][\mathrm{Cl}}^{-}] \end{array}$$

Scheme BC:

(37) $$\begin{array}{l} \frac{d[{\mathrm{ClO}}_{3}^{-}]}{dt}=-k_{5}[{\mathrm{ClO}}_{3}^{-}{][\mathrm{HClO}}_{2}]+k_{3B}{\left[{\mathrm{HClO}}_{2}\right]}^{2};\\ \frac{d[{\mathrm{ClO}}_{2}]}{dt}=2k_{5}[{\mathrm{ClO}}_{3}^{-}{][\mathrm{HClO}}_{2}]-k_{4}[{\mathrm{ClO}}_{2}]+2k_{3B}{\left[{\mathrm{HClO}}_{2}\right]}^{2}+4k_{3C}[{\mathrm{HClO}}_{2}{][\mathrm{Cl}}^{-}];\\ \frac{d[{\mathrm{HClO}}_{2}]}{dt}=-k_{5}[{\mathrm{ClO}}_{3}^{-}{][\mathrm{HClO}}_{2}]+k_{4}[{\mathrm{ClO}}_{2}]-4k_{3B}{\left[{\mathrm{HClO}}_{2}\right]}^{2}-5k_{3C}[{\mathrm{HClO}}_{2}{][\mathrm{Cl}}^{-}];\\ \frac{d[{\mathrm{Cl}}^{-}]}{dt}=k_{3B}{\left[{\mathrm{HClO}}_{2}\right]}^{2}+k_{3C}[{\mathrm{HClO}}_{2}{][\mathrm{Cl}}^{-}] \end{array}$$

Scheme KG:

(38) $$\begin{array}{l} \frac{d[{\mathrm{ClO}}_{3}^{-}]}{dt}=-k_{5}[{\mathrm{ClO}}_{3}^{-}{][\mathrm{HClO}}_{2}]+k_{3B}{\left[{\mathrm{HClO}}_{2}\right]}^{2};\\ \frac{d[{\mathrm{ClO}}_{2}]}{dt}=2k_{5}[{\mathrm{ClO}}_{3}^{-}{][\mathrm{HClO}}_{2}]-k_{4}[{\mathrm{ClO}}_{2}]+2k_{3B}{\left[{\mathrm{HClO}}_{2}\right]}^{2}+\frac{4k_{3C}[{\mathrm{HClO}}_{2}{][\mathrm{Cl}}^{-}{]}^{2}}{K+[{\mathrm{Cl}}^{-}]};\\ \frac{d[{\mathrm{HClO}}_{2}]}{dt}=-k_{5}[{\mathrm{ClO}}_{3}^{-}{][\mathrm{HClO}}_{2}]+k_{4}[{\mathrm{ClO}}_{2}]-4k_{3B}{\left[{\mathrm{HClO}}_{2}\right]}^{2}-\frac{5k_{3C}[{\mathrm{HClO}}_{2}{][\mathrm{Cl}}^{-}{]}^{2}}{K+[{\mathrm{Cl}}^{-}]};\\ \frac{d[{\mathrm{Cl}}^{-}]}{dt}=k_{3B}{\left[{\mathrm{HClO}}_{2}\right]}^{2}+\frac{k_{3C}[{\mathrm{HClO}}_{2}{][\mathrm{Cl}}^{-}{]}^{2}}{K+[{\mathrm{Cl}}^{-}]}; \end{array}$$

In conformity with the balance relation for the total concentration of Cl atoms in solution the sum of all temporal derivatives of all Cl-containing components is equal to zero for all kinetic schemes: (34), (35) and (36):

(39) $$\frac{d[{\mathrm{ClO}}_{3}^{-}]}{dt}+\frac{d[{\mathrm{ClO}}_{2}]}{dt}+\frac{d[{\mathrm{HClO}}_{2}]}{dt}+\frac{d[{\mathrm{Cl}}^{-}]}{dt}=0,\;i.e.\;[{\mathrm{ClO}}_{3}^{-}]+[{\mathrm{ClO}}_{2}]+[{\mathrm{HClO}}_{2}]+[{\mathrm{Cl}}^{-}]=\mathrm{const}$$

Initial condition for the chlorate concentration at *t* = 0 (starting moment of electrolysis):

(40) $$\left[{{\mathrm{ClO}}_{3}}^{-}\right]\;=\;{\left[{{\mathrm{ClO}}_{3}}^{-}\right]}^{0}≅\left[{\mathrm{NaClO}}_{3}\right]=50\;mM$$

since the fraction of the decomposed chlorite in the course of the solution preparation is very small. The very first spectroscopic measurement in the course of the electrolysis provides “the starting value of [ClO_2_]”, [ClO_2_]^0^ = 0.08 mM. However, contrary to a reliable estimate for [ClO_3_^−^]^0^ (which is very close to [NaClO_3_] = 50 mM), the ClO_2_ concentration, [ClO_2_], changes rapidly within the initial time interval. Additionally, the chemical decomposition of chlorate generates *various* Cl-containing components subjected to rapid reactions between them, which may not be described by the general scheme of kinetic Equations (34)–(36). It means that the above “initial experimental value of [ClO_2_], i.e., [ClO_2_]^0^, is known *not quite reliably*. This point will be discussed in more detail in the course of the further theoretical procedure; see below.

### 2.6. Further Theoretical Analysis

Further theoretical analysis including the determination of the values of all unknown parameters of the system (*k*_5_, *k*_3A_/*k*_3B_/*k*_3C_, initial concentrations, etc.) from comparison of calculated plots with experimental data was carried out with the use of *dimensionless* variables:

(41) $$t*=k_{4}t,\;y_{5}=\frac{\left[{\mathrm{ClO}}_{3}^{-}\right]}{\left[{\mathrm{NaClO}}_{3}\right]},\;y_{4}=\frac{\left[{\mathrm{ClO}}_{2}\right]}{\left[{\mathrm{NaClO}}_{3}\right]},\;y_{3}=\frac{{[\mathrm{HClO}}_{2}]}{\left[{\mathrm{NaClO}}_{3}\right]},\;y_{1}=\frac{{[\mathrm{Cl}}^{-}]}{\left[{\mathrm{NaClO}}_{3}\right]}$$

i.e., dimensionless time, t*, is introduced with the use of an *already found* value of the *k*_4_ rate constant: *k*_4_ = 5.2 × 10^−4^ s, Equation (10), while all time-dependent concentrations are normalized via division by the initial chlorate concentration, [NaClO_3_] ≅ 50 mM, Equation (40).

First of all, the above set of kinetic Equations (34)–(38) for time-dependent concentrations of all solute Cl-containing components is presented in the dimensionless form (Section S3 of Supplementary Information). The method to obtain a numerical solution of these differential equations for each set of the system’s parameters is described in Section S4. The fitting procedure that allowed us to determine the optimal values of the fitting parameters is exposed in Section S5.

The results of application of this fitting procedure are described below for various kinetic schemes: A, B, C, BC and KG, corresponding to different expressions for the kinetics of the chlorous acid (HClO_2_) disproportionation step.

## 3. Comparison of Predictions of Various Models with Experimental Data Graphical Illustrations

### 3.1. Scheme A

This mechanism is based on the HClO_2_ disproportionation on the platinum surface via the reaction scheme (17) as a catalytic heterogeneous process, with generation of chlorine dioxide, ClO_2_, and chloride anion, Cl^−^ [65,66]. Its kinetics is described by Equation (18). The kinetics of the global process in dimensionless variables is given by the set of Equations (S6).

**Figure 6:**
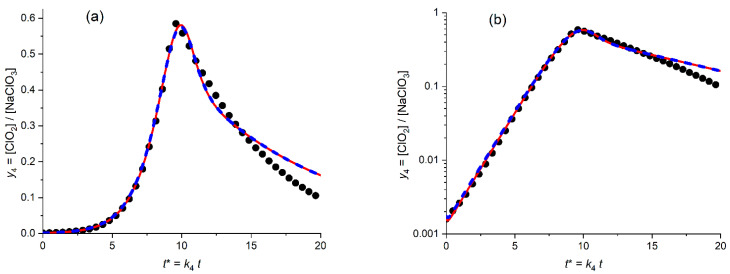
Scheme A. Comparison between experimental data for the dimensionless ClO_2_ concentration evolution (black points, see Figure 5d) and its theoretical simulation (red solid and blue dash lines) calculated for the optimal values of *two* kinetic dimensionless parameters: *K*_54_ and *K*_34A_ at a fixed initial value of the ClO_2_ concentration equal to its experimental one: *y*_4_^0^ = 1.6 × 10^−3^ (red line) or of *three* fitting parameters: *K*_54_, *K*_34A_ and *y*_4_^0^ (blue line) in the *linear* coordinates: *y*_4_ vs. t* (**a**) or in the *semi-logarithmic* coordinates: log *y*_4_ vs. t* (**b**). Initial value of HClO_2_ concentration: *y*_3_^0^ = 0. Optimization procedure (see Section S5) is carried out in *linear* coordinates, i.e., with the use of the SV^abs^ function defined by Formula (S15) in Section S5.

Figure 6a presents the calculated plot (red solid line) for the temporal evolution of the dimensionless ClO_2_ concentration, *y*_4_ = [ClO_2_]/*c*_5_^0^, for the *optimized* values of the fitting parameters (kinetic constants, *K*_54_ = 6.8, *K*_34A_ = 0.18) in comparison with the experimental plot (points) in the *linear* coordinates, *y*_4_^(exper)^ vs. t*. This search of the *optimal* values of the parameters was performed via the minimization procedure of the mean-square distance in the *linear* coordinated (Figure 6a), i.e., with the use of the SV^abs^ function defined by Formula (S15) in Section S5.

One can see that the calculated line reproduces properly the whole shape of the experimental plot, which possesses obvious *autocatalytic* features: a drastic growth in the catalytic (ClO_2_) component starting from very low values, with the passage through a narrow maximum and a subsequent rapid decrease. The optimal values of the fitting parameters allow one to reach a good agreement between the calculated and experimental values of the dimensionless coordinates of the maximum, i.e., of its time, t*_max_ (9.9 vs. 9.6) and of the maximal concentration, *y*_4,max_ (0.58 vs. 0.59) One can also see that the increasing branch before passing the maximum is also well simulated by the theory, while there is an evident disparity between the calculated and experimental plots within the decreasing branch.

The same data are shown in Figure 6b in the *semi-logarithmic* coordinates, i.e., log *y*_4_ vs. t*. This second graph is able to provide an *extra useful information*. First of all, one can see that kinetic scheme A is able to reproduce the behavior of the experimental plot *within the initial stage of the global process* where the ClO_2_ concentration is still *close to its initial value*, *y*_4_^0^. One should take into account that the *initial* value of the ClO_2_ concentration, i.e., *y*_4_^0^ was *not* among the fitting parameters, i.e., it was equal to the *experimental* value, 1.6 × 10^−3^. Therefore, the observed agreement in Figure 6b within the small time interval should be expected.

Figure 6b also shows that the calculated line in these coordinates reproduces well an *almost linear dependence within a very broad time range of the concentration increase*. On the contrary, the decreasing branch of the calculated line *deviates strongly upward from a linear behavior* while the experimental dependence is much closer to a linear one.

In order to check on whether such a disparity between theoretical and experimental plots within a long time range might be lift out via a broader choice of the fitting parameters, their number was increased via the addition of initial values of dimensionless concentrations, *y*_i_^0^, in particular, the one of the chlorine dioxide, *y*_4_^0^.

The effect of its variation is shown as a blue line in Figure 6a,b. Effectively, the red and blue theoretical curves in Figure 6 superimpose almost perfectly. This overlap is a direct consequence of the optimal values of the corresponding parameters, which are very close to each other, e.g., the optimal value of *y*_4_^0^ is around 0.17 × 10^−3^ for blue lines in Figure 6a,b (instead of 1.6 × 10^−3^ for red lines).

Thus, one can conclude as a whole that kinetic scheme A is *unable to describe properly the long-time behavior* of the experimental data.

### 3.2. Scheme B

This mechanism (as well as all other mechanisms considered below) is based on the HClO_2_ disproportionation *inside the solution phase*. Its specific feature is the primary step, which is the rate-determining one and which includes a reaction between two HClO_2_ molecules so that its rate to proportional to ([HClO_2_])^2^, i.e., *y*_3_^2^ (see Equation (20)), and the full set of kinetic Equations (S7) in the dimensionless form.

**Figure 7:**
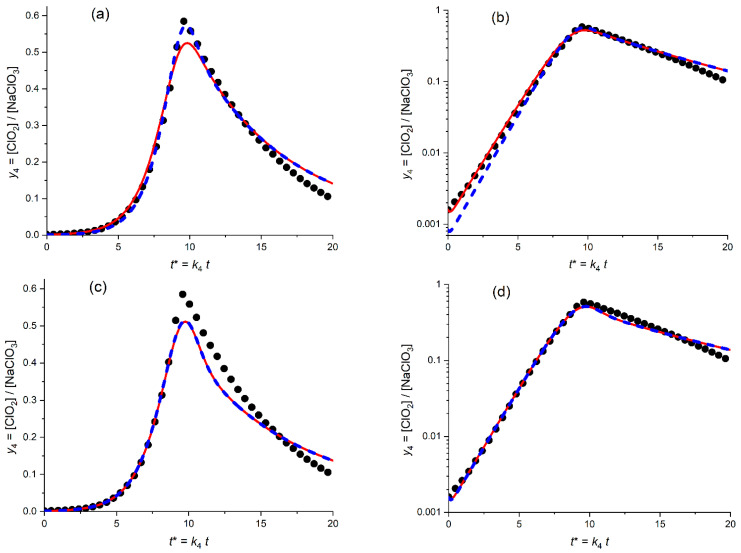
Scheme B. Comparison between experimental data for the dimensionless ClO_2_ concentration evolution (black points, see Figure 5d) and its theoretical simulation (red solid and blue dash lines) calculated for the optimal values of *two* kinetic dimensionless parameters: *K*_54_ and *K*_34B_ at a fixed initial value of the ClO_2_ concentration equal to its experimental one: *y*_4_^0^ = 1.6 × 10^−3^ (red line), or of *three* fitting parameters: *K*_54_, *K*_34B_ and *y*_4_^0^ (blue line) in the *linear* coordinates: *y*_4_ vs. t* (**a**,**c**) or in the *semi-logarithmic* coordinates: log *y*_4_ vs. t* (**b**,**d**). Initial value of HClO_2_ concentration: *y*_3_^0^ = 0. Optimization procedure (see Section S5) is carried out in *linear* (**a**,**b**) or *semilogarithmic* (**c**,**d**) coordinates.

Figure 7a presents the calculated plot (red solid line) for the temporal evolution of the dimensionless ClO_2_ concentration, *y*_4_ = [ClO_2_]/*c*_5_^0^, for the *optimized* values of the fitting parameters (kinetic constants, *K*_54_ = 4.7, *K*_34B_ = 0.78) in comparison with the experimental plot (points) in the *linear* coordinates, *y*_4_^(exper)^ vs. t*.

One can see that even though the calculated line reproduces properly the *general* shape of the experimental plot with its *autocatalytic* features: a drastic growth of the catalytic (ClO_2_) component starting from very low values, with the passage through a narrow maximum and a subsequent rapid decrease as well as the time moment of the concentration maximum, t*_max_. However, the quantitative agreement is obviously *worse* than that for scheme A (Figure 6a), even within the growing branch of the plot as well as for the maximal concentration, *y*_4,max_ (the theoretical maximum is evidently lower). The disparity within the starting segment of the decreasing branch is weaker than that in Figure 6a, but the theoretical plot does not reproduce properly experimental data within the long time range either.

The blue dashed lines in Figure 7a,b present similar results for the fitting procedure with variation of *three* parameters: two kinetic ones, *K*_54_ and *K*_34B_, as well as the initial ClO_2_ concentration, i.e., *y*_4_^0^. This change diminishes markedly the distance between the theoretical and experimental data in Figure 7a, which is reached first of all due to their close proximity near the *maximum* of the plots. On the other hand, the blue dash line fully repeats the deviation of the red solid line from experimental points on the descending branch in Figure 7a.

Similar analysis in the semi-logarithmic coordinates (Figure 7b, blue dash line) reveals that the *improvement near the maximum* is counterbalanced by drastic *deviation* of the line from experimental points within the *initial time range*.

Figure 7c,d present the results for the theoretical plots found via the SV^relative^ fitting procedure, Equation (S17) in Section S5, for *two* or *three* varying parameters (red solid line or blue dashed line, respectively). First of all, one can conclude that (unlike the SV^abs^ fitting in Figure 7a,b discussed above) both theoretical lines in Figure 7c,d practically coincide with one another since the optimal value of the varying *y*_4_^0^ parameter, 1.57 × 10^−3^) is very close to the experimental one, 1.6 × 10^−3^. As a whole, the theoretical and experimental plots in Figure 7d look pretty close within the growing region and near the maximum, while there is a marked deviation within the decreasing region. However, a comparison of Figure 7a,c shows evidently that theoretical plots found via the SV^relative^ fitting procedure are *much worse* compared to those in Figure 7a, both in the vicinity of the maximum and within the decreasing branch.

### 3.3. Scheme C

This mechanism is again based on the HClO_2_ disproportionation *inside the solution phase*, this time in the presence of a significant amount of chloride anion, Cl^−^. Then, this species plays the role of *a catalyst*: at the primary (rate-determining) step. Namely, it reacts with chlorous acid with formation of a complex, which is transformed into the reaction products, Equation (17), including the “catalytic” chloride ion, after a set of rapid chemical steps. As a result, the HClO_2_ disproportionation rate is proportional to the product of HClO_2_ and Cl^−^ concentrations, i.e., to *y*_3_ and *y*_1_ (see Equation (21)), and the full set of Equations (S8).

A specific feature of integration procedure in this case is the necessity to choose a *nonzero initial value of the chloride anion*, i.e., *y*_1_^0^, since the experimental value of *y*_1_^0^ is unknown while for *its zero* value both the rate of the HClO_2_ disproportionation and the Cl^−^ concentration would be equal to zero at any moment of the process.

**Figure 8:**
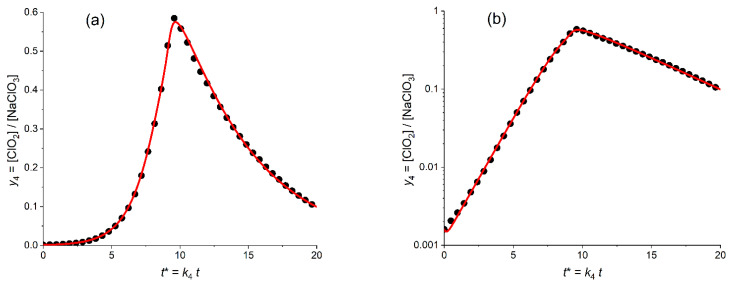
Scheme C. Comparison between experimental data for the dimensionless ClO_2_ concentration evolution (black points, see Figure 5d) and its theoretical simulation (red solid line) calculated for the optimal values of *three* fitting dimensionless parameters: *K*_54_, *K*_34C_ and *y*_1_^0^ at a fixed initial value of the ClO_2_ concentration equal to its experimental one: *y*_4_^0^ = 1.6 × 10^−3^ in the *linear* coordinates: *y*_4_ vs. t* (**a**) or in the *semi-logarithmic* coordinates: log *y*_4_ vs. t* (**b**). Initial value of HClO_2_ concentration: *y*_3_^0^ = 0. Optimization procedure (see Section S5) is carried out in *linear* coordinates.

This is why one has to start from the fitting procedure for *three varying parameters*: *K*_54_, *K*_34C_ and *y*_1_^0^. The results for the *linear* and *semi-logarithmic* coordinates are given in Figure 8a,b, respectively.

Figure 8a,b present the calculated plot (solid line) for the temporal evolution of the dimensionless ClO_2_ concentration, *y*_4_ = [ClO_2_]/*c*_5_^0^, for the *optimized* values of the fitting parameters (kinetic constants, *K*_54_ = 4.4, *K*_34C_ = 29.5 as well as the initial value: *y*_1_^0^ = 2.3 × 10^−5^) in comparison with the experimental plot (points) in the *linear* coordinates, *y*_4_^(exper)^ vs. t* (a) and in the *semi-logarithmic* coordinates, log *y*_4_^(exper)^ vs. t* (b). The initial value of the ClO_2_ concentration, i.e., parameter *y*_4_^0^ has been chosen in conformity with its experimental value, 1.6 × 10^−3^.

Both figures, as well as the very small values of the distances between the optimal theoretical and experimental plots, SV^abs^ and SV^relative^, testify in favor of their very close proximity.

The distance between the theoretical and experimental plots in Figure 8a could be further diminished via the fitting procedure with inclusion of the *varying* value of *y*_4_^0^, i.e., with *four* fitting parameters. It results in the diminution of the *optimal* value of *y*_4_^0^ to around 1.3 × 10^−3^, with a slight increase in the kinetic parameters. However, such a change in the *y*_4_^0^ value would lead inevitably to a *noticeably worse* agreement in the *semi-logarithmic* coordinates, while “a closer agreement in the linear coordinates” does not shift the modified theoretical plots compared to that in Figure 8a.

Despite this *very good* reproduction of experimental data within the whole time range by the calculated lines in Figure 8a,b, on the basis of scheme C, one cannot conclude that the corresponding set of kinetic Equations (36) and (S8) for the concentrations of Cl-containing species gives a definitive description of the chlorate reduction process. One should be reminded that scheme C is based on expression (21) for the HClO_2_ disproportionation step, which is *valid only in the presence of a sufficiently large Cl^−^ concentration,* while this condition is *violated within the initial time range* since the values of its initial concentration, *y*_1_^0^, found in the course of the fitting (from 10^−5^ to 10^−4^) are *very low*. As a result, the contribution of term (21) into the whole ClO_2_ disproportionation rate for such low Cl^−^ concentrations is *practically negligible* compared to the calculated values of another contribution to the rate of this step given by expression (20) for its rate constant: *k*_3B_ = 0.29 × 10^−2^ M^−1^ s^−1^, found experimentally (see above). This means that it is *incorrect to neglect by this contribution B*, as it is made within the framework of scheme C.

In other words, one has to conclude that a substantiated description of the chlorate reduction process within the whole time range must include both contributions, i.e., originated both from B and from C mechanisms. Thus, it should be based on scheme BC or scheme KG.

### 3.4. Scheme BC

Let us start from a simpler variant: scheme BC. This mechanism is again based on the HClO_2_ disproportionation *inside the solution phase*, while this reaction proceeds via *two parallel mechanisms* described above as schemes B and C. They start from the HClO_2_ reactions either with another HClO_2_ molecule (scheme B) or with Cl^−^ anion (scheme C), respectively, where these primary steps are *rate-determining*. This approximation leads to expression (22) for the rate of the HClO_2_ disproportionation and, consequently, to the sets of Equation (37).

This mechanism is again based on the HClO_2_ disproportionation *inside the solution phase,* while this reaction proceeds via *two parallel mechanisms* described above as schemes B and C, i.e., starting from the HClO_2_ reactions either with another HClO_2_ molecule or with Cl^−^ anion, respectively, where these primary steps are *rate-determining*. This approximation leads to expression (22) for the rate of the HClO_2_ disproportionation and, consequently, to the sets of Equation (37).

Its dimensionless variant, Equation (S9), contains six parameters: three combinations of kinetic constants, *K*_54_, *K*_34B_ and *K*_34C_, as well as the initial values of the concentrations, *y*_4_^0^ (ClO_2_), *y*_3_^0^ (HClO_2_) and *y*_1_^0^ (Cl^−^). From the analyses of schemes A, B and C, one may expect that the values of *y*_3_^0^ and *y*_1_^0^ are sufficiently small so that one can start the consideration from their *zero* value while the value of *y*_4_^0^ is known from experimental data (1.6 × 10^−3^). As for the kinetic constants, the value of *K*_34B_ is known from the experimental study of the HClO_2_ disproportionation process [73], see Equation (S11): *K*_34B_ = 0.28 (being independent of pH). On the contrary, the pH-dependent parameter, *K*_34C_, has only been measured for much lower acid concentrations: *K*_34C_ is equal to 0.30 for 1.2 M HClO_4_ and to 0.58 for 2 M HClO_4_. It means that one can only conclude that its value should be *much larger than 0.6* because of a much higher proton activity in the 8 M H_2_SO_4_ solution in this study.

Thus, as the first stage of this analysis for scheme BC let us *minimize the number of fitting parameters* via fixing the above value of *K*_34B_ as well as all initial concentrations, i.e., by varying only two kinetic parameters: *K*_54_ and *K*_34C_.

The optimization procedure for the *linear* coordinates, i.e., minimization of SV^abs^, Equation (S15), gives the values of the fitting parameters: *K*_54_ ≅ 4.6, *K*_34C_ ≅ 21. In conformity with intuitive expectations the former parameter, *K*_54_, is close to that for scheme C while *K*_34C_ *has diminished markedly* from 29.5. The latter effect is evidently a consequence of the necessity to compensate *a positive contribution from the B term* into the rate of the HClO_2_ disproportionation, Equation (22).

One may note that the found value of *K*_34C_ for the 8 M H_2_SO_4_ solution is in conformity with the expectations that it should be *much larger than those for solutions of 1 m to 2 M acid concentrations* (0.3–0.6). One cannot give a more quantitative prediction since the effect is probably related to the *proton activity*, *a*(H^+^), rather than to its *concentration,* while the former grows much faster than the latter within this concentration range (this effect has been found earlier for the bromate reduction process [34]). Additionally, the kinetic order for the relation between *K*_34C_ and *a*(H^+^) is not known. Finally, the process is significantly accelerated *at higher ionic strength* [34,73].

**Figure 9:**
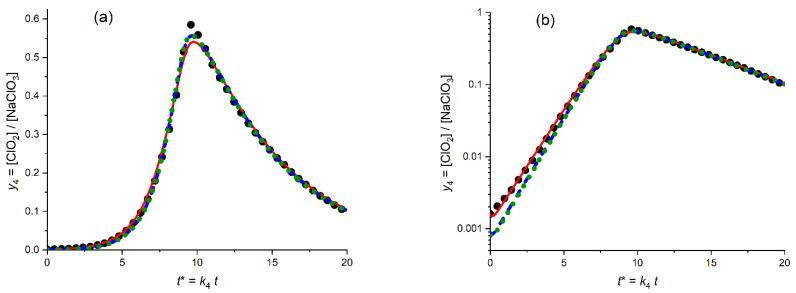
Scheme BC. Comparison between experimental data for the dimensionless ClO_2_ concentration evolution (black points, see Figure 5d) and its theoretical simulation (red solid, blue dash and green dotted lines) calculated for the optimal values of *two* kinetic dimensionless parameters: *K*_54_ and *K*_34C_ at fixed initial values of the concentrations equal to its experimental one for ClO_2_: *y*_4_^0^ = 1.6 × 10^−3^ and *y*_1_^0^ = 0 (red line), or of *three* fitting parameters: *K*_54_, *K*_34C_ and *y*_4_^0^ at fixed initial values of other concentrations: *y*_1_^0^ = 0 (blue line), or of *four* fitting parameters: *K*_54_, *K*_34C_, *y*_4_^0^ and *y*_1_^0^ (green line) in the *linear* coordinates: *y*_4_ vs. t* (**a**) or in the *semi-logarithmic* coordinates: log *y*_4_ vs. t* (**b**). Initial value of HClO_2_ concentration: *y*_3_^0^ = 0. Optimization procedure (see Section S5) is carried out in *linear* coordinates.

Comparison between the corresponding theoretical plot (red solid line in Figure 9a) and the experimental one (black points in Figure 9a) shows that the former line reproduces well not only the general features of the latter one but also both the increasing and decreasing branches as well as the maximal time, t*_max_. The only region where some deviation is visible is around the maximum, where the calculated line *underestimates* the maximal ClO_2_ concentration: *y*_4,max_ is equal to 0.54 vs. 0.58 experimentally.

Analogous analysis in the *semi-logarithmic* coordinates is given in Figure 9b. Similar to Figure 8b for scheme C, one may conclude on *a uniformly close proximity between the calculated and experimental plots*. The good agreement within the small time region is a direct consequence of the choice of *y*_4_^0^ as equal to its experimental value.

It has also been studied whether the distance between the calculated and experimental plots *in the vicinity of the maximum* might be diminished owing to variation of both the kinetic parameters, *K*_54_ and *K*_34C_, and the *initial value of the ClO_2_ concentration, y*_4_^0^ (blue line in Figure 9), or the *initial values of ClO_2_ and Cl^−^ concentrations*, *y*_4_^0^ and *y*_1_^0^ (green line in Figure 9)

These blue and green lines in Figure 9a demonstrate that the differences between the maximal values of the ClO_2_ concentration in the *linear* coordinates has become *almost twice smaller*: 0.56 vs. 0.585 while the agreement between the plots in the other time regions *visually* remained similarly well so that the value of the distance between plots, SV^abs^, *diminished by a factor of two*. However, the analysis in the *semi-logarithmic* coordinates (Figure 9b, blue and green lines) revealed *a strong deviation of the theoretical plot within the short-time region*. This divergence of the lines is a consequence of an *improper* choice of the initial ClO_2_ concentration: *y*_4_^0^ is around 0.9 × 10^−3^ (blue line) and 0.8 × 10^−3^ (green line) instead of 1.6 × 10^−3^. One can see from Figure 9b that this deviation of the predicted concentration from its experimental value remains significant within almost the whole time range of the concentration increase. Since one cannot accept a marked deviation of the calculated plot for the ClO_2_ concentration from the experimental one within *an extended time region,* one has *to reject* the attempt to get a better reproduction of the vicinity of the maximum owing to variation of the initial ClO_2_ concentration. The absence of visible distinctions between the blue and green curves in Figure 9, even with non-equal numbers of optimized parameters, originates from a very small difference in y_1_^0^ values: 0 (blue line) versus 1 × 10^−5^ (green line).

The fitting procedure has also been carried out for the intermediate variant where the value of *y*_4_^0^ has been fixed (being equal to its experimental value, 1.6 × 10^−3^) while the distance between the plots has been minimized via variation of *three* parameters: *K*_54_, *K*_34C_ and the *initial value of the Cl^−^ concentration*, *y*_1_^0^. Thus, the optimal values of the kinetic parameters have turned out to be *practically identical to those without variation of the initial concentration*, i.e., at *y*_1_^0^ = 0, with the same behavior of the plots around its maximum in both linear and semi-logarithmic coordinates.

Thus, one may conclude that scheme BC has provided a quantitative agreement between its predictions and experimental data within *almost* whole time range (Figure 9a,b), except for the region in the vicinity of the maximum where the difference between the calculated and experimental values of the ClO_2_ concentration reaches 8%.

### 3.5. Scheme KG

This mechanism is based on the same information for the HClO_2_ disproportionation process *inside the solution phase* (identical to that for scheme BC), in particular, on its passage via *two parallel mechanisms* described above as schemes B and C, i.e., starting from the HClO_2_ reactions either with another HClO_2_ molecule or with Cl^−^ anion, respectively.

The dimensionless variant of Equations (24) and (38), i.e., Equation (S10), now contains seven parameters: four combinations of kinetic constants, *K*_54_, *K*_34B,_ *K*_34C_ and *K*_C_, as well as the initial values of the concentrations, *y*_4_^0^ (ClO_2_), *y*_3_^0^ (HClO_2_) and *y*_1_^0^ (Cl^−^). Similar to the analysis of scheme BC, one may expect that the values of *y*_3_^0^ and *y*_1_^0^ are sufficiently small so that one can start the consideration from their *zero* values, while the value of *y*_4_^0^ is known from experimental data (1.6 × 10^−3^). As for the kinetic constants, the values of *K*_34B_ and *K*_C_ are known from the experimental study of the HClO_2_ disproportionation process [73], see Equation (S11): *K*_34B_ = 0.28, *K*_C_ = 0.024 (being independent of pH). As for the pH-dependent kinetic constant, *K*_34C_, it has only been measured for much lower acid concentrations: *K*_34C_ is equal to 0.30 for 1.2 M HClO_4_ and to 0.58 for 2 M HClO_4_. One should note that a much larger value: *K*_34C_ ≅ 21 has been obtained for scheme BC in 8 M H_2_SO_4_ solution.

**Figure 10:**
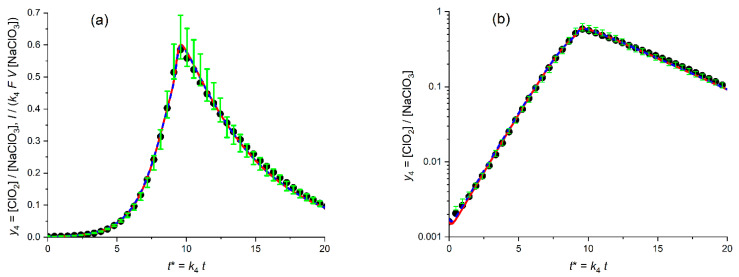
Scheme KG. Comparison between experimental data for the dimensionless ClO_2_ concentration evolution (black points, see Figure 5d) and its theoretical simulation (red solid and blue dash lines) calculated for the optimal values of *two* kinetic dimensionless parameters: *K*_54_ and *K*_34C_ at fixed initial values of the concentrations equal to its experimental one for ClO_2_: *y*_4_^0^ = 1.6 × 10^−3^ and *y*_1_^0^ = 0 (red line), or of *three* fitting parameters: *K*_54_, *K*_34C_ and *y*_4_^0^ (blue line) in the *linear* coordinates: *y*_4_ vs. t* (**a**) or in the *semi-logarithmic* coordinates: log *y*_4_ vs. t* (**b**). Initial values of HClO_2_ and Cl^−^ concentrations: *y*_3_^0^ = 0, *y*_1_^0^ = 0. Optimization procedure (see Section S5) is carried out in *linear* coordinates. (**a**) also presents experimental data the current in the form: *I*/(*k*_4_ *F V* [NaClO_3_]) (green points with indication of the upper and lower limits due to rapid fluctuations of the current, see Figure 5e.

Thus, as the first stage of this analysis for scheme KG, let us *minimize again the number of fitting parameters* via fixing the above values of *K*_34B_ and of *K*_C_ as well as all initial concentrations, i.e., by varying only two kinetic parameters: *K*_54_ and *K*_34C_. Results of the fitting procedure are presented as red lines in Figure 10 in the linear (a) and semi-logarithmic (b) coordinates.

**Figure 11:**
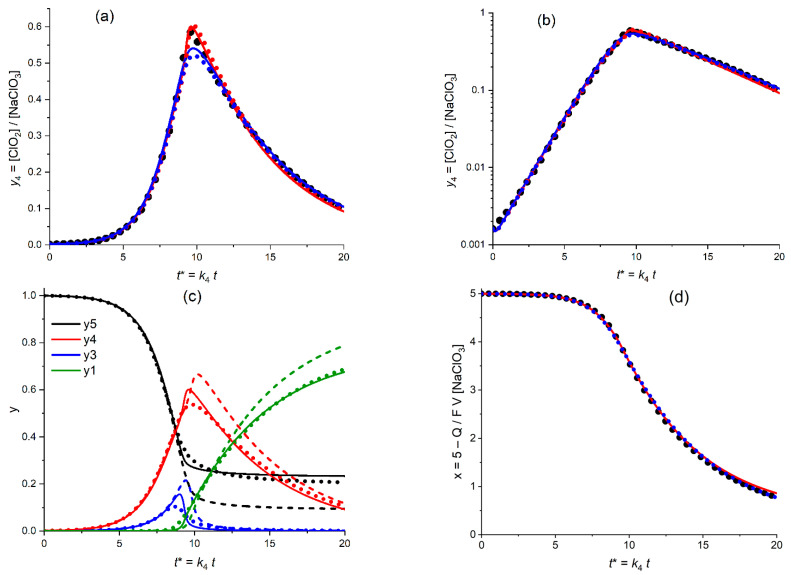
Schemes BC and KG. (**a**,**b**) Theoretical plots for the ClO_2_ concentration evolution for optimized values of two kinetic parameters: *K*_54_ = 4.4, *K*_34C_ = 55 for min SV^abs^ (red solid line) or *K*_54_ = 4.3, *K*_34C_ = 48 for min SV^relative^ (red dotted line) for Scheme KG; *K*_54_ = 4.6, *K*_34C_ = 21 for min SV^abs^ (blue solid line) or *K*_54_ = 4.4, *K*_34C_ = 22 for min SV^relative^ (blue dotted line) for Scheme BC in comparison with experimental data (black points) in the linear coordinates: *y*_4_ vs. t* (**a**) or in the semi-logarithmic coordinates: log *y*_4_ vs. t* (**b**). Initial values of HClO_2_ and Cl^−^ concentrations: *y*_3_^0^ = 0, *y*_1_^0^ = 0. (**c**) Evolution of the concentrations of all Cl-containing components: ClO_3_^−^ (black lines), ClO_2_ (red lines), HClO_2_ (blue lines) and Cl^−^ (green lines); optimized values of the kinetic parameters for Scheme BC (dotted and dash lines) and for Scheme KG (solid lines); kinetic Equation (22) (dotted lines) or (24) (dash and solid lines). (**d**) Evolution of the average oxidation degree of Cl atoms in solution, *x*, related to the normalized passed reduction charge: *x* = 5 − *Q*/(*F V* [NaClO_3_]): experimental data (black points), predictions for Scheme BC (blue line) or for Scheme KG (red line).

One can conclude an almost perfect agreement of calculated KG plots with experimental data in both coordinates for the whole time range from the beginning of the process up to the middle of the decreasing branch, in particular, including the vicinity of the maximum where the proximity has improved significantly compared to that for scheme BC; see Figure 11 below for more detail.

The attempt to reach a closer agreement with experimental data via variation of the initial ClO_2_, i.e., *y*_4_^0^ (besides two kinetic parameters, *K*_54_ and *K*_34C_) changes only slightly its optimal value (to 1.7 × 10^−3^), compared to the experimental one (1.6 × 10^−3^). As a consequence two- and three-parameter theoretical lines (red and blue ones) for Scheme KG are practically *overlapping* both in Figure 10a,b.

Comparison of the optimal values of the *three* fitting parameters for Schemes BC and KG shows that those of *K*_54_ and *y*_4_^0^ belong for both schemes to narrow intervals: between 4.2 and 4.4 or between 1.6 × 10^−3^ and 1.75 × 10^−3^ due to the additional small term, *K*_C_, in the denominator of expression (24) for Scheme KG. On the other hand, this additional term, *K*_C_, has resulted in a noticeable increase in the value of *K*_34C_ from 21 for BC to about 55 for KG. Moreover, both values *are about two decimal orders of magnitude larger* than that found experimentally in [73] for lower acidities. This enormous increase in *K*_34C_ has been expected in view of a much larger acid amount (8 M H_2_SO_4_ in our studies vs. 1.2 M and 2 M in [73]) and consequently a higher ionic strength and much higher proton activity. At the same time, a very good agreement with experimental data in Figure 10 has been achieved with *the use of the values of two pH-independent parameters*, *k*_1_ and *K*, *measured in the earlier study of the HClO_2_ disproportionation* [73].

Experimental data for the current are also added in Figure 10a,b both for the linear and for semi-logarithmic coordinates, with indication of *its oscillations’ amplitude* which originate from fluctuations of the hydrodynamic field inside the cell due to the pump functioning. We have already drawn on the basis of Figure 5d that each experimental point for the HClO_2_ concentration (its fluctuations are much weaker) falls into the range of the current dispersion. It leads to the conclusion on the proportionality between the current and the ClO_2_ concentration within the whole time range (except for the very beginning of the process where the points in Figure 5c deviate slightly upwards). It means that the chlorine dioxide reduction is *the only electrochemical step of the chlorate reduction process*. Figure 10a,b provide us with another important observation on the agreement between the calculated theoretical line of the ClO_2_ concentration evolution with the passing non-stationary current within the whole time range of the process, as is predicted by its kinetic scheme.

### 3.6. Temporal Evolution of the Concentrations and of the Average Cl-Atom Oxidation Degree

Theoretical plots for Schemes KG (red solid and dotted lines) and BC (blue lines/points) are presented in Figure 11a,b for the optimal values of two kinetic parameters, K54 and K34C which (for each scheme). These values are found by minimization:

- Either of SV^abs^, Equation (S15), i.e of the distance of the corresponding theoretical *line* from the experimental plot (black points) in the *linear* coordinates (Figure 11a);
- Or of SV^relative^, Equation (S17), i.e of the distance of the corresponding theoretical *point line* from the experimental plot (black points) in the *semi-logarithmic* coordinates (Figure 11b).

Comparison of these theoretical plots for *the same scheme* (solid and dotted lines of the same color) demonstrates a very slight difference between them in the vicinity of the maximum in Figure 11a (*solid* lines being systematically *closer to experimental data*) while no visible difference may be found for the lines in Figure 11b. Thus, one can conclude: if minimizations of SV^abs^ and SV^relative^ give *identical* (or *very close*) values of *y*_4_^0^ which are *identical to its experimental value* (if this parameter is not varied), or these values are *very close to its experimental one, the optimal values of the kinetic parameters should be found by minimization of* SV^abs^. On the contrary, if thus found values of *y*_4_^0^ are *significantly different from its experimental value*, then such theoretical plots will reveal *an inacceptable deviation from experimental data* in the *semi-logarithmic* coordinates.

This conclusion has already been used above in Figure 6, Figure 7, Figure 8, Figure 9 and Figure 10 in the course of the choice of the optimal parameters.

As a consequence of the choice of the initial value of the ClO_2_ concentration (*y*_4_^0^ is equal to its experimental value) all plots in Figure 11b (for Schemes BC and KG as well as for min SV^abs^ or min SV^relative^) are practically overlapping both with each other and with experimental data, with slight deviations within a relatively short time interval and near the maximum.

On the contrary, these four plots can be clearly distinguished in the *linear* coordinates (Figure 11a). These differences between the lines (for min SV^abs^ or min SV^relative^) for *the same scheme* are relatively small while the separation of lines *for different schemes* (KG vs. BC) is significant within two time ranges. This effect is especially strong *within the vicinity of the maximum* where *the proximity between the theoretical KG line for min SV^abs^ and experimental data is excellent* while the maximal ClO_2_ concentrations for both BC lines are *markedly lower*. An opposite deviation takes place *within the very-long time range* where both BC plots overlap perfectly with experimental data while the KG lines are shifted slightly downwards. As a whole, the standard deviation between theoretical and experimental ClO_2_ concentration plots, SV^abs^, calculated for the optimized values of the varied parameters for Scheme KG (see cation to Figure 11) has diminished markedly (by 10% to 20%) both for Figure 11a and for Figure 11b.

Comparison of the theoretical plots for Schemes KG and BC (Figure 11a,b) has revealed their *unexpectedly large difference* in the vicinity of their maxima: the relative difference between the maximal theoretical and experimental ClO_2_ concentrations is about 8% for Scheme BC while it practically *vanishes* for Scheme KG.

Extra information on the origin of this strong effect is provided by the calculated plots for the evolution of concentrations of all Cl-containing components (Figure 11c). For each concentration there are three plots: dotted and solid lines correspond to the Equation (S9) or (S10) for the values of parameters, *K*_54_ and *K*_34C_, optimized for Scheme BC or Scheme KG while dash lines represent solutions of Equation (S10) with parameters optimized for Scheme BC.

Thus, comparison between dash and dotted lines represents in a pure form the effect of the *additional* parameter, *K*_C_ = 0.024, in the denominator of expression (SI10), for *the same* values of the other fitting parameters: *K*_54_ = 4.57, *K*_34C_ = 21.2, *y*_4_^0^ = 1.6 × 10^−3^ found for Scheme BC, i.e., for *K*_C_ = 0. One can see from Figure 11c that the deviation of a dash line from the corresponding dotted one starts first of all from the *blue* lines (around t* ≅ 7) where the values of *y*_3_ (HClO_2_) are about 0.1 while the values of *y*_1_ (Cl^−^, green lines) are still *very small*: *K*_C_ ≫ *y*_1_. Within this time region the rate of the HClO_2_ transformation (term *v_3_*_C_) for Scheme BC is *much greater* than that for Scheme KG. As a result, the blue dotted line for Scheme BC *passes through a maximum*, with the further monotonous decrease to zero; it leads to the diminution of the chlorate transformation rate (black dotted line) and to the passage of ClO_2_ concentration through a maximum (red dotted line). On the contrary, within this time region the HClO_2_ transformation rate for Scheme KG is *much slower* so that the HClO_2_ concentration (*y*_3_, blue dash line) *continues its rapid growth*; it results in the continuation of a *rapid consumption of chlorate ion* (*y*_5_, black dash line) via the comproportionation step, accompanied by *a rapid growth of ClO_2_* (*y*_4_, red dash line), thus reaching *a much higher maximum*: up to 0.666 for dash line instead of 0.538 for dotted line, i.e., increase over 22%.

This drastic change is significantly reduced by the opposite effect because of the change of optimal values of the parameters due to the transition from Scheme BC to Scheme KG. As a result, the increase in the maximal ClO_2_ concentration diminishes up to about 10% (instead of 22%), as it is seen from the red solid line. An additional KG parameter, K_C_, is also responsible for the specific shape of this plot (drastic increase at red solid line) within the time region just prior the maximum passage. On the other hand, after the passage of their maxima both ClO_2_ and HClO_2_ concentrations diminish faster for Scheme KG than for Scheme BC while the effect of a strong increase in the total chlorate transformation degree (black dahs line: from about 80% to the limit over 90%) is even (slightly) overcompensated (black solid line).

As one can see from Figure 11d, there is no marked difference at all in the predictions for the evolution of the average oxidation degree of Cl atoms in the system: *x* = 5 − *Q/*( *F V* [NaClO_3_]), between Schemes BC and KG. These theoretical predictions reproduce perfectly the experimental plot, with a very slight upward deviation within the most extended time range.

The use of Scheme KG (similar to Scheme BC) for description of the HClO_2_ disproportionation process has got an important advantage that it takes into account the well-established information on existence of *two parallel mechanisms* of this process so that its rate is described by a sum of two corresponding contributions, Equation (24). The first term gives the dominant contribution *within the short-time range* (increasing branch) where the second contribution is small due to a very low Cl^−^ ion concentration while the HClO_2_ concentration is relatively high (Figure 11c). In the region around the maximum of the plots the second contribution grows rapidly in parallel to the Cl^−^ concentration so that it becomes the dominant one.

## 4. Materials and Methods

Content of this section is available in Supplementary Information (Section S1).

## 5. Conclusions

(1) This study has confirmed *unique properties* of the EC-autocat mechanism based on the transformation of an *electrochemically inert* component (e.g., chlorate which does not react at an electrode within the studied potential range) via a *redox-mediator cycle* composed of electrochemical and chemical steps *if the cycle possesses autocatalytic features*. It means that *its passage results in increase in the number of catalytic species*, i.e., components of the redox couple based on *the same chemical redox-active element*, e.g., Cl in the catalysis of ClO_3_^−^ reduction via the ClO_2_/HClO_2_ redox couple. Namely, *even without addition of these catalytic species into the starting solution* the rate of the global process can reach *high values,* which are only limited by the initial amount of the principal component (e.g., chlorate). Such a surprising behavior is due to a quasi-exponential increase in the concentrations of these catalytic species owing to proceeding of the redox cycle, even if their initial amount may be very low. For example, a sufficient amount of ClO_2_ is automatically generated via slight decomposition of chlorate anion in the course of the starting solution preparation.
(2) In the case of the chlorate reduction process in a strongly acidic medium, *the key role in its transformation* is played by the components of the ClO_2_/HClO_2_ redox couple. Within its redox-mediator cycle, ClO_2_ is *easily reduced* at an electrode (even at a non-modified carbon one) while its reduction product in acidic media, HClO_2_, can be subjected to *the comproportionation step with chlorate ion*. Such a combination of stages makes the redox cycle *autocatalytic*. This mechanism has already been proposed (without a *quantitative analysis* of the process) in [52,53,54,55] as a basis for the steady-state production of chlorine dioxide from chlorate in an acidic solution *electrochemically*.
(3) Our studies of chlorate electroreduction have been oriented towards its application for *power sources*. In this context, the above mechanism composed of one electrochemical and one chemical step would *not* be prospective since chlorate would be reduced *finally* up to HClO_2_, i.e., with the transfer only of *two* electrons per Cl atom which means a *low redox-charge density* of the chlorate solution: *only one third* of that for the *six-electron* chlorate-to-chloride transformation. From this point of view the existence of the *second chemical step*, i.e., the *HClO_2_ disproportionation* represents *a very important advantage* for *a drastic increase in the redox-charge and energy densities* of the chlorate reagent, owing to *generation of the chloride anion, Cl^−^, by this reaction*, as a final product of the global process.
(4) In this context, the kinetics of the global chlorate process has been analyzed for 5 previously proposed variants of the HClO_2_ disproportionation, with comparison of their theoretical predictions with our experimental data for the evolution both of the current and of the ClO_2_ concentration in the course of the chronoamperometric study with synchronous spectrophotometric analysis of electrolyte. It has been established that the *quantitative* interpretation of these data can be achieved on the basis of the kinetic mechanism where the ClO_2_ electroreduction and ClO_3_^−^+HClO_2_ comproportionation steps are combined with the HClO_2_ disproportionation one, the latter step taking place via *two parallel routes* where its total reaction rate is described by the Kieffer-Gordon expression [73]. Our study has confirmed the values of the pH-independent parameters of this expression while those of the pH-dependent parameters (in particular, the rate constant of the ClO_3_^−^+HClO_2_ comproportionation) have been determined for 8 M H_2_SO_4_ solution for the first time.
(5) The substantiated model of the global chlorate-to-chloride reduction process has allowed us to establish the temporal evolution of the concentrations of all Cl-containing components: ClO_3_^−^, ClO_2_, HClO_2_ and Cl^−^, as well as of the total redox charge of the solution and of the average oxidation degree of Cl atoms in the course of the reductive electrolysis.
(6) The proposed model (including the found values of its kinetic parameters) may be immediately used in the course of an analysis of the chlorate anion electroreduction in acidic media:
  - For estimation of the maximal current that can be generated by the chlorate-based power source as a function of initial composition of the chlorate-acid solution;
  - For calculation of the ClO_2_ concentration evolution, including its maximal value as well as the time needed to reach this maximum, etc.

We believe that the proposed set of kinetic equations containing already determined rate constants of the chemical stages may be employed for other regimes of the chlorate-reduction process inside an electrochemical cell, e.g., for the galvanostatic regime where a proper expression the rate of the electrochemical stage is to be used. Moreover, this approach is applicable for the description of this process inside the positive-electrode compartment of *a power source* based on the chlorate reduction where it will allow one to predict its principal characteristics, e.g., its faradaic and energy efficiencies.

## Data Availability

The data presented in this study are available on request from the corresponding authors.

## Supplementary Materials

The following supporting information can be downloaded at: https://www.mdpi.com/article/10.3390/molecules30163432/s1, File S1: SUPPLEMENTARY INFORMATION; Mechanism and kinetics of non-electroactive chlorate electroreduction via catalytic redox-mediator cycle without catalyst’s addition (EC-autocat process).
