# Supplementary material for: Mechanism and Kinetics of Non-Electroactive Chlorate Electroreduction via Catalytic Redox-Mediator Cycle Without Catalyst’s Addition (EC-Autocat Process)
Source: Molecules. 2025 Aug 20;30(16):3432. doi: 10.3390/molecules30163432 (PMC12388821; doi:10.3390/molecules30163432)
Supplement: Supplementary file 1 [file molecules-30-03432-s001.zip › molecules-3714490-supplementary.pdf]

SUPPLEMENTARY INFORMATION

# Mechanism and kinetics of non-electroactive chlorate electroreduction via catalytic redox-mediator cycle without catalyst's addition (EC-autocat process)

Mikhail A. Vorotyntsev [1,*], Pavel A. Zader [1,*], Olga A. Goncharova [1,2] and Dmitry V. Konev [1,2]
email addresses: mivo2010@yandex.com, paul.zadyor@gmail.com, goncharovaooolga@gmail.com, dkfrvzh@yandex.ru

[1] Frumkin Institute of Physical Chemistry and Electrochemistry Russian Academy of Sciences, 119071 Leninsky prospekt 31-4, Moscow, Russia
[2] Federal Research Center for Problems of Chemical Physics and Medicinal Chemistry Russian Academy of Sciences, 142432 N.N. Semenov's avenue 1, Chernogolovka, Moscow Region, Russia

Corresponding authors:
*e-mail: mivo2010@yandex.com (M.A. Vorotyntsev)
**e-mail: paul.zadyor@gmail.com (P.A. Zader)

## S1. Materials and Methods

Chlorate solutions in acidic medium as electrolyte for performing reductive electrolysis were prepared from concentrated sulfuric acid ($H_2SO_4$, chemically pure, Himmed, Moscow, Russia) and sodium chlorate ($NaClO_3$, 99+%, Acros Organics, Fair Lawn, USA). Weighed portion of the solid salt powder was dissolved in sulfuric acid solution preliminary prepared on the basis of tridistillate water, with subsequent water addition up to certain volume. Such a procedure allows us to minimize the chlorate chemical decomposition due to both the heat effect because of a significant acid dilution and the contact of solute chlorate with more concentrated acid solution in the course of its preparation. Freshly prepared solutions have only been used for each electrochemical experiment.

Home-made special setup (Figure S1) has been designed and constructed to attain the maximal degree of the chlorate electroreduction. Its main component is represented by electrochemical cell with divided compartments where counter electrodes (1) are separated from the working-electrolyte space (2) by electrolytic keys (3). Working electrode: circular part of platinum plate (4) (diameter: 17 mm) in contact with chlorate solution inside cylindrical chamber (5); plate (4) covers its bottom with the use of sealed flange joint (6). Potential of the working electrode is measured *vs*. Ag/AgCl reference electrode in contact with saturated KCl solution (7) (its potential is equal to 0.198 V *vs*. SHE). All values of the electrode potential in the manuscript are given versus this Ag/AgCl reference electrode. Circuit (8) of flow-through spectrophotometric cuvette (9) is connected through walls of chamber (5) where circulation of electrolyte is driven by peristaltic pump (10). Flow of electrolyte via circuit (8) is needed not only to analyze spectrophotometrically the variation of the mediator concentration in the electrolyte but also to maintain uniform distribution of its components inside the electrolyte throughout the whole working space of the cell. In addition, the solution circulation ensures conditions for functioning of the working electrode with a constant mass transfer coefficient in the oncoming jet mode.

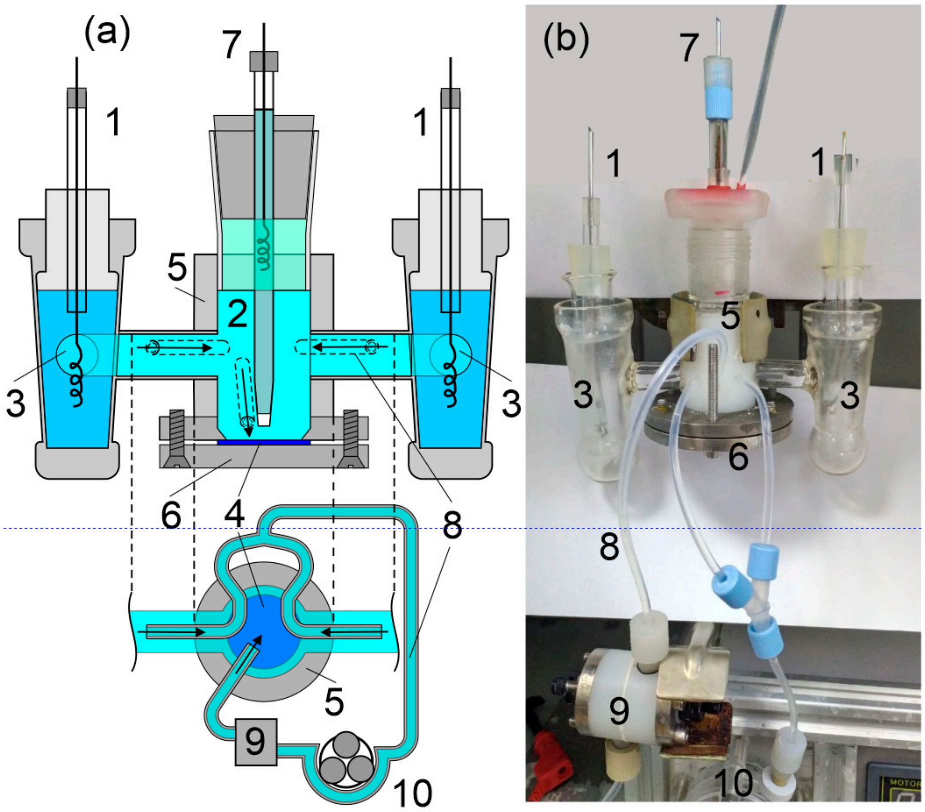


Figure S1. (a) Scheme of experimental setup to carry out electrolysis of the sulfur-acidic solution of sodium chlorate with parallel spectrophotometric analysis of the electrolyte composition inside the working compartment. Below: fragment of the cell from above and circuit for solution circulation through the spectrophotometric cuvette connected to it; (b) photograph of the setup. See the text above for explanation of notations

The used peristaltic pump produces inevitably an *unsteady flux of the solution* via circuit (8), with short-time oscillations around a mean value of the flux. It leads to a subsequent variation of the convection intensity inside the electrochemical cell and consequently in the fluctuative variation of both the non-stationary diffusion layer thickness near the electrode surface and the diffusion-limited current. The latter looks in experimental chronoamperograms (see e.g. Figures 1a, 2a, 3a, 5b) as a *widened line for the current*. In Figures 5c,d,e and 10a the chronoamperogram of Figure 5b is represented as a set of *I,t* points with indication of amplitudes of these local current fluctuations as a *dispersion* of the current. These fluctuations of the hydrodynamic field and consequently of the diffusion-limited $ClO_2$ flux to the electrode surface should lead to fluctuations of the averaged $ClO_2$ concentration inside the cell but their amplitude is much smaller (because of a slow relaxation of the concentration distribution inside the internal region of the diffusion boundary layer) so that they are not visible in Figure 5a.

Two counter electrodes (1) were made of strips of platinum foil (dimensions: ~ 2x15 mm); they were in electric contact with current supply via platinum wire sealed in glass. To polarize the working electrode, they were both connected via the current supply to the corresponding terminal of the potentiostat, thus providing their parallel connection to the circuit of the current passage through the working electrode. This connection is used to diminish the ohmic resistance of electrolytic keys

(3) in the polarizing circuit. In the course of the electrolysis process the counter electrodes were immersed in sulfuric acid solution of the same concentration as that in the working solution; the cathodic current through the working electrode passes via counter electrodes owing to the process of oxygen release due to water oxidation.

Proceeding of electrochemical experiments: cathode (5) and both anode chambers (3) were filled with aqueous solution of sulfuric acid (6 M or 8 M); electrolytic keys (3) were locked. Circulation of solution (2) through the working chamber along circuit (8) was started and the baseline for flow cell (9) was recorded. Background acid solution in cathode chamber (5) was replaced with a precisely measured volume (about 10 mL) of sulfuric solution (the same acid concentration) of sodium chlorate (50 – 200 mM), prepared immediately prior to experiment in volumetric flask. Circulation of this solution through cell (9) was started at a constant rate of about 4 volumes per minute (i.e. 40 mL/min). Periodic registration of the spectra of the working solution (by means of fiber-optic spectrophotometer Avantes Avaspec 2048 (Avaspec ULS2048CL-EVO-RS, Avantes B.V., Netherlands) was started synchronously with the step of the working electrode (4) potential, with current measurement in the potentiostatic mode (potentiostat P-50, Electrochemical Instruments, Russia). It gives both a chronoamperogram of the chlorate electroreduction from a sulfuric acid solution and a set of UV-visible spectra of this solution (with intervals of 10 s) in the course of the whole duration of the process. The procedure for treatment of the measured set of spectra in order to extract data on the evolution of the chlorine dioxide concentration is described below in section **S2**.

## S2. Determination of the $ClO_2$ concentration evolution on the basis of spectral measurements

Set of spectra of the solution in the course of the chronoamperometric study for the potential step up to 0.7 V (black line for the current in Figure 3a) is given in Figure S2 which is identical to Figure 4 of the manuscript.

Within the initial time range the absorption within a broad band around 360 nm (related to solute $ClO_2$) rapidly increases (Figure S2a), up to the absorption saturation within the medium range of the band. After the moment when the current passes through its maximum (black line in Figure 3a) the intensity of the solution absorption decreases (Figure S2b).

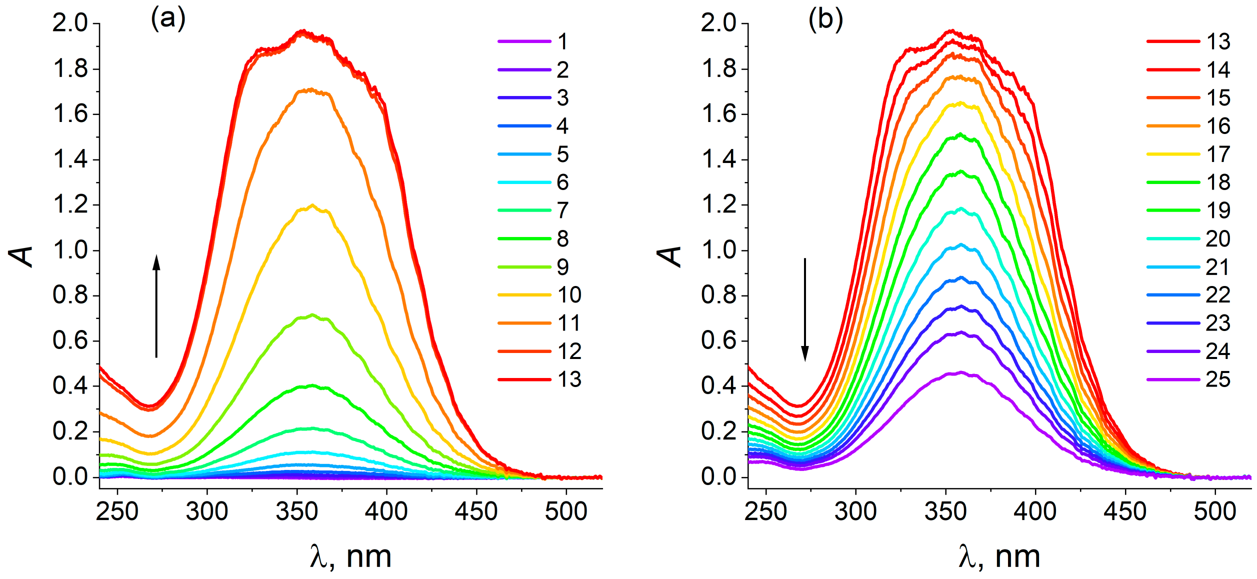


Figure S2. Evolution of the registered UV-visible spectrum of the electrolyte solution in the course of the chronoamperometric measurement for potential step to 0.7 V (current evolution: black line in Figure 3a): time range of increasing current and absorption, lines 1 to 13 (a); time range of decreasing current and absorption, lines 13 to 25 (b). Background absorption (average value of the absorbance within the range between 500 nm and 520 nm) has been subtracted from each spectrum

Chlorine dioxide concentrations for the set of time moments have been determined from the spectral data in Figure S2 within the wavelength range between 358 nm and 430 nm where the absorption is solely related to $ClO_2$. Principally, this operation is based on the Buger–Lambert–Beer law on the proportionality between the absorbance at a wavelength, $A(\lambda,\mathrm{N})$, and the concentration, $C(\mathrm{N})$, of the absorbing species, $ClO_2$, for spectrum N:

$$A(\lambda,\mathrm{N}) = \varepsilon(\lambda)\, L\, C(\mathrm{N}) \tag{S1}$$

where $L$ is the optical path length ($L$ = 0.0854 cm), $\varepsilon(\lambda)$ is the extinction coefficient at this wavelength, N is the number of the spectrum indicated in the list in Figure S2. However, this relation is only valid within the *linearity region* of the absorption. Therefore, one has first of all to establish *the limit of this region*.

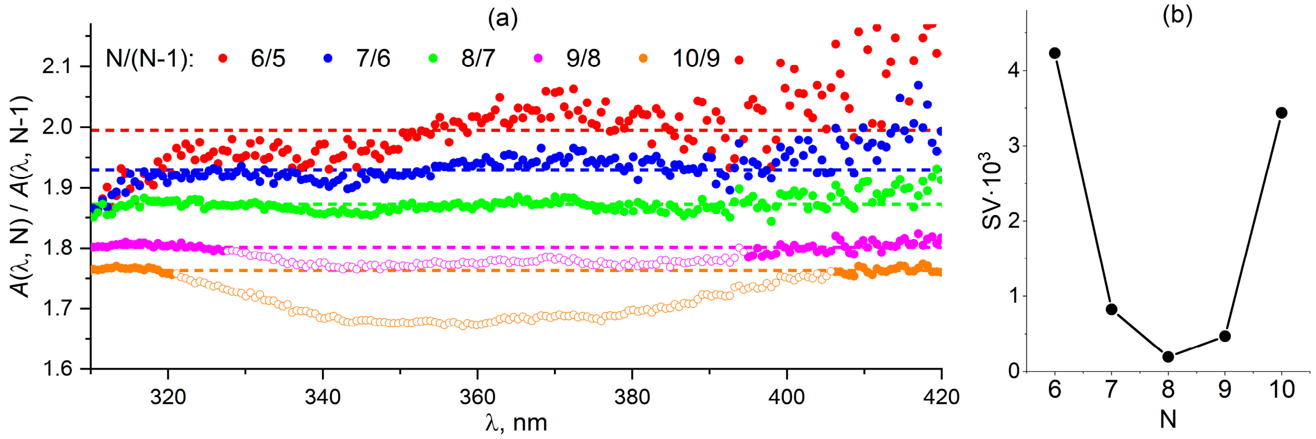


Figure S3. (a) Plots for the ratios of absorbances (at each wavelength, λ) for two subsequent spectra in Figure S2: $A(\lambda,\mathrm{N})$ / $A(\lambda,\mathrm{N}\text{-}1)$; values of N/(N-1) are indicated for each dotted line in legend; each

horizontal dash line shows the average value of the corresponding line within the wavelength region between 320 nm and 420 nm; (b) mean-square distances, SV(N), of the experimental points of the corresponding ratios in Figure S3a from their average values within this wavelength region for $6 \leq N \leq 8$ and within the wavelength regions where $A(\lambda,N) < 0.5$ for N = 9 and N = 10 (see the text below for details)

Plots for the ratios of absorbances (at each wavelength, λ) for two subsequent spectra: $A(\lambda,N) / A(\lambda,N-1)$ for several values of the N number (indicated for various spectra in Figure S2) from 6 to 10 are shown as dotted lines in Figure S3a. For the range of the wavelengths where linear relation (S1) is *valid for both spectra,* N-1 and N, this ratio is *equal to the ratio of the corresponding concentrations*:

$$A(\lambda,N) / A(\lambda,N-1) = C(N) / C(N-1), \qquad (S2)$$

i.e. *the ratio is independent of the wavelength.*

One can see that within a broad wavelength region between 320 nm and 420 nm which includes the whole upper part of the $ClO_2$ absorption band (see Figure S2) the constancy condition, (S2), is satisfied in Figure S3a for N = 8 (green line) within limits of the experimental noise. It means that linearity relation (S1) is satisfied within the whole wavelength range for *both* spectra of the ratio, i.e. for $\boldsymbol{A(\lambda,7)}$ and $\boldsymbol{A(\lambda,8)}$. For the previous ratios, in particular, for N = 7 (blue line) and N = 6 (red line) this constancy condition is less evident because of a much larger noise-to-signal relation, while there is no *global* deviation of these ratios from the constancy, too. In any case the linearity condition is surely satisfied for all N < 8 since their absorbances are weaker than that for the plot at N = 8.

One can see quite a different behavior for *larger* N values in Figure S3a, i.e. for N = 9 (magenta line) and especially for N = 10 (marron line) which correspond to *larger absorbances* (Figure S1) and correspondingly to *higher $ClO_2$ concentrations*. For these plots in Figure S3a *a pronounced well* appears within a broad wavelength region *centered around the absorption band maximum*: $\lambda_{max}$ = 358.5 nm. The depth of this well *increases monotonously for higher $ClO_2$ concentrations* (Figure S3a).

For the sake of a greater clearness the magenta and marron plots are shown in Figure S3a *as empty circles within the well region* while these plots are given *as sets of filled circles outside the well region* where the linearity condition is satisfied. The *horizontal thin dash lines* for these plots represent *the averaged values* of the corresponding plots *outside their well regions*, thus making the well depth reliably visible.

Appearance of these wells and variation of their depth in the series of plots in Figure S3a is an obvious manifestation of the *non-linearity effect* which *decreases* the absorbance, *A*, in comparison with predictions of linear relation (S1): $A_{non-linear}(\lambda,N) < A_{linear}(\lambda,N)$. It is the central region around

$\lambda_{max}$ where the solution absorbance (for the same $ClO_2$ concentration) grows most rapidly so that this effect appears first of all within this region. Since the absorbance for the wavelength is markedly stronger for the $A(\lambda,N)$ spectrum, compared to that for the $A(\lambda,N-1)$ one, then the ratio of the *non-linear absorbances*: $A_{non\text{-}linear}(\lambda,N)$ / $A_{non\text{-}linear}(\lambda,N-1)$, is *less* than the ratio of the *linear absorbances*: $A_{linear}(\lambda,N)$ / $A_{linear}(\lambda,N-1)$, resulting in a well around the band maximum. Besides, the effect *increases* in parallel with the N value. It is exactly what is observed in Figure S3a for N = 9 (magenta line) and especially for N = 10 (marron line).

Since the analysis of the green plot for N = 8 has already proven *the applicability of linear relation (S1) towards* A(λ,8) the *non-linearity effect* revealed in the plot for $A(\lambda,9)$ / $A(\lambda,8)$ should be attributed to the numerator, $A(\lambda,9)$, and consequently to $A(\lambda,10)$.

The above derivation of the *linearity region at the absorption maximum* is based on *visible* observations in Figure S3a. As a supplementary evidence let us consider mean-square distances (SV) of the experimental points of the corresponding plots in Figure S3a from their average values (shown as thin dash lines in Figure S3a) within the wavelength region between 320 nm and 420 nm for several N values (Figure S3b). This graph reveals *a very deep minimum of SV* at N = 8 while its value *increases drastically both for smaller N values* (because of much stronger noise-to-signal ratio) and *for larger N values* (because of the appearance of a well of an increasing depth within the top of the absorption band, due to the *non-linearity effect*).

As a result, one can conclude that near the maximum of the principal absorption band of $ClO_2$ ($\lambda_{max}$ = 358.5 nm [67,76]) *the linearity conditions are already markedly violated* starting from N = 9 since *an evident well is visible within this range*. It means that one *cannot determine the $ClO_2$ concentrations* from relation (S1) for $N \geq 9$ at $\lambda_{max}$ while the values of the extinction coefficient, ε, are not available for other wavelengths.

On the contrary, for smaller N values ($N \leq 8$) the *non-linearity effect may be neglected inside a broad wavelength interval which includes the vicinity of the band maximum*. Thus, one can use formula (S1) at $N \leq 8$ to find the $ClO_2$ concentration for each of these spectra with the use of the maximal extinction value: $\varepsilon(\lambda_{max})$ =1250 $M^{-1}$ $cm^{-1}$ [67,76] and $L$ = 0.0854 cm, in particular:

$$C(1) = 0.081 \text{ mM}, C(8) = 3.9 \text{ mM} \quad \text{(S3)}$$

Thus, because of a large value of the extinction coefficient of $ClO_2$ at the maximum of its absorption band linear relation (S1) between the absorbance and the $ClO_2$ concentration is only valid within a *low* concentration range (below 5 mM) where the absorbance, $A$, for the optical cuvette and the spectrum measurement system used for this study (section S1 above) does not exceed markedly 0.5.

Since the spectral data at $\lambda_{max}$ for N ≥ 9 are *useless because of a too high absorbance* one has to use such data *for longer wavelengths* where the absorbance (for the same value of N) may be *much lower* because of a smaller extinction.

Then, one needs *to determine their extinction values* with the use of another useful formula (S4) for the ratio of the absorbance values at *two different wavelengths*, $\lambda_1$ and $\lambda_2$, for *the same spectrum*, N, which is also valid only *under the linearity conditions*:

$$A(\lambda_1,N) / A(\lambda_2,N) = \varepsilon(\lambda_1) ./ \varepsilon(\lambda_2) \quad (S4)$$

Relation (S4) has been applied first of all for $\lambda_1$ = 410 nm, $\lambda_2 = \lambda_{max}$ where the maximal concentration *inside the linearity range*, i.e. at N = 8 (to minimize fluctuations-to-absorbance ratio). The use of the absorbance values, $A(\lambda,8)$, at $\lambda_{max}$ and at 410 nm (Figure S2) as well as relation (S4) gives for the extinction coefficient at 410 nm: ε(410 nm) = 475 $M^{-1}$ $cm^{-1}$.

Since this value at 410 nm is much lower than that at $\lambda_{max}$ while the saturation limits for the absorbances at these two wavelengths are relatively close to one another (according to Figure S2), *the linearity region for 410 nm is strongly extended towards to larger N numbers*, i.e. *to higher concentrations*, as it is visible in Figure S4a. On the other hand, due to the same reason the subregion of N numbers where the absorbance is *too weak* because of *too strong noise* is also extended. Both effects become even stronger for λ = 420 nm and especially for λ = 430 nm (Figure S4a.).

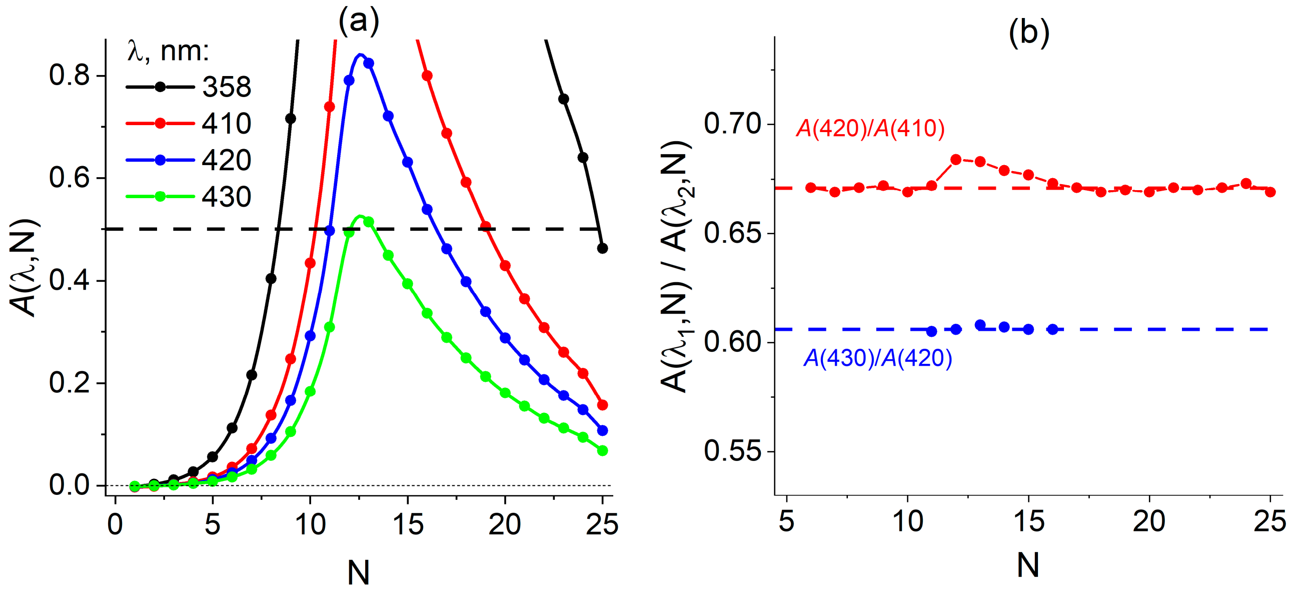


Figure S4. (a) Dependences of absorbances, *A*, on the spectrum's number, N (see Figure S2) at several wavelengths: $\lambda_{max}$ = 358.5 nm, 410 nm, 420 nm and 430 nm; horizontal dash line shows the linearity limit. (b) Dependences of the ratios of absorbances at *two different wavelengths*, $\lambda_1$ and $\lambda_2$: $A(\lambda_1,N) / A(\lambda_2,N)$, for *the same spectrum*, N. Red line: $\lambda_1$ = 410 nm, $\lambda_2$ = 420 nm; blue line: $\lambda_1$ = 420 nm, $\lambda_2$ = 430 nm

In Figure S4b the values of the ratio: $A(\lambda_1,N) / A(\lambda_2,N)$, are plotted for $\lambda_1 = 420$ nm, $\lambda_2 = 410$ nm (red points) and for $\lambda_1 = 430$ nm, $\lambda_2 = 420$ nm (blue points) as functions of the spectrum number, N. The points where the absorbance in the numerator is already too weak have been disregarded: red points for N < 6 and blue points for N < 11 and for N > 16 (in conformity with data in Figure S4a).

The ratio for 420-vs-410 nm (red points) has turned to be *practically constant* within the range of N from 6 to 25, except for the solutions of the highest $ClO_2$ concentrations (N from 12 to 15) where the absorbances at 420 nm and especially at 410 nm becomes well above the assumed linearity limit, 0.5 (see Figure S4a).

The values of $A$(430 nm) are much smaller than those for 420 nm (see Figure S4a); it is the reason why the range of spectrum numbers for the 430-to-420 nm plots is limited to a few points, only, where the noise effect becomes sufficiently small. In the middle of this N range one can see again *a maximum (of a small amplitude, though) due to the non-linearity effect* because of too large absorbance values for 420 nm.

In view of relation (S4) the dash lines in Figure S4b provide the values of the ratios of extinctions: ε(420 nm) / ε(410 nm) and ε(430 nm) / ε(420 nm). Taking into account the above found value of ε(410 nm), it gives the values of two extinction coefficients: ε(420 nm) = 332.5 $M^{-1}$ $cm^{-1}$, ε(430 nm) = 213.5 $M^{-1}$ $cm^{-1}$.

Thus found parameters have allowed us to calculate the concentrations for all spectra in Figure S2 with the use of absorbances at the wavelength: $A$(358.5 nm), or $A$(410 nm), or $A$(420nm), or $A$(430 nm), so that the absorbance does not exceed the same upper limit of the linearity region, 0.5-0.6. This procedure has provided us with the set of $ClO_2$ concentrations for all spectra in Figure S2.

The result in the form of the $ClO_2$ concentration as a function of the electrolysis time is presented in Figure 5a of the paper. In particular, within the increasing branch the concentration changes *over several decimal orders of magnitude*, from about 0.08 mM up to almost 30 mM at the maximum. After passing the maximum the concentration diminishes monotonously.

## S3. Set of kinetic equations for concentrations of Cl-containing components in dimensionless variables

Dimensionless variables for time and the concentrations of solute Cl-containing components have been introduced by formulae:

$$t^* = k_4 t, \quad y_5 = \frac{[ClO_3^-]}{[NaClO_3]}, \quad y_4 = \frac{[ClO_2]}{[NaClO_3]}, \quad y_3 = \frac{[HClO_2]}{[NaClO_3]}, \quad y_1 = \frac{[Cl^-]}{[NaClO_3]} \qquad (S5)$$

Equations (34), (35), (36) and (37) take the form of relations for the *dimensionless concentrations*, $y_5$, $y_4$, $y_3$ and $y_1$ as functions of *dimensionless time*, $t^*$:

Scheme A:

$$\frac{dy_5}{dt^*} = -K_{54}y_5y_3; \quad \frac{dy_4}{dt^*} = 2K_{54}y_5y_3 - y_4 + 4K_{34A}y_3;$$
$$\frac{dy_3}{dt^*} = -K_{54}y_5y_3 + y_4 - 5K_{34A}y_3; \quad \frac{dy_1}{dt^*} = K_{34A}y_3 \qquad \text{(S6)}$$

Scheme B:

$$\frac{dy_5}{dt^*} = -K_{54}y_5y_3 + K_{34B}(y_3)^2; \quad \frac{dy_4}{dt^*} = 2K_{54}y_5y_3 - y_4 + 2K_{34B}(y_3)^2;$$
$$\frac{dy_3}{dt^*} = -K_{54}y_5y_3 + y_4 - 4K_{34B}(y_3)^2; \quad \frac{dy_1}{dt^*} = K_{34B}(y_3)^2 \qquad \text{(S7)}$$

Scheme C:

$$\frac{dy_5}{dt^*} = -K_{54}y_5y_3; \quad \frac{dy_4}{dt^*} = 2K_{54}y_5y_3 - y_4 + 4K_{34C}y_3y_1;$$
$$\frac{dy_3}{dt^*} = -K_{54}y_5y_3 + y_4 - 5K_{34C}y_3y_1; \quad \frac{dy_1}{dt^*} = K_{34C}y_3y_1 \qquad \text{(S8)}$$

Scheme BC:

$$\frac{dy_5}{dt^*} = -K_{54}y_5y_3 + K_{34B}(y_3)^2; \quad \frac{dy_4}{dt^*} = 2K_{54}y_5y_3 - y_4 + 2K_{34B}(y_3)^2 + 4K_{34C}y_3y_1;$$
$$\frac{dy_3}{dt^*} = -K_{54}y_5y_3 + y_4 - 4K_{34B}(y_3)^2 - 5K_{34C}y_3y_1; \quad \frac{dy_1}{dt^*} = K_{34B}(y_3)^2 + K_{34C}y_3y_1 \qquad \text{(S9)}$$

Scheme KG:

$$\frac{dy_5}{dt^*} = -K_{54}y_5y_3 + K_{34B}(y_3)^2;$$
$$\frac{dy_4}{dt^*} = 2K_{54}y_5y_3 - y_4 + 2K_{34B}(y_3)^2 + \frac{4K_{34C}y_3y_1^2}{K_C + y_1};$$
$$\frac{dy_3}{dt^*} = -K_{54}y_5y_3 + y_4 - 4K_{34B}(y_3)^2 - \frac{5K_{34C}y_3y_1^2}{K_C + y_1}; \qquad \text{(S10)}$$
$$\frac{dy_1}{dt^*} = K_{34B}(y_3)^2 + \frac{K_{34C}y_3y_1^2}{K_C + y_1}; \text{ where } K_C = \frac{K}{c_5^0} = 0.024$$

These equations for each scheme contain *two* (Schemes A, B and C), or *three* (Scheme BC), or even *four* (Scheme KG) dimensionless parameters:

$K_{54} = (k_5 / k_4)$ [NaClO$_3$] for all schemes as well as $K_{34A} = k_{3A} / k_4$ for Scheme A, $K_{34B} = (k_{3B} / k_4)$ [NaClO$_3$] for Schemes B and BC,

or $K_{34C} = (k_{3C} / k_4)$ [NaClO$_3$] for Schemes C or BC, or $K_{34C} = (k_{3C} / k_4)$ [NaClO$_3$] and $K_C = K$ / [NaClO$_3$] for Scheme KG, respectively.

Some of these dimensionless parameters can be calculated for Schemes BC and KG from the values of the pH-independent kinetic parameters: $k_1 = 1.17\ 10^{-2}$ M$^{-1}$ s$^{-1}$, $K = 1.2$ mM while $k_2$ is equal to 1.57 $10^{-2}$ M$^{-1}$ s$^{-1}$ for [H$^+$] = 1.2 M and 3.00 $10^{-2}$ M$^{-1}$ s$^{-1}$ for [H$^+$] = 2 M [73] and the initial chlorate concentration, [NaClO$_3$] $\cong$ 50 mM so that

$$K_{34B} = \left(\frac{k_1}{4k_4}\right)[NaClO_3] = 0.28, \quad K_C = \frac{K}{[NaClO_3]} = 2.4\times10^{-2} \quad \text{(independent of pH)}$$
$$K_{34C} = \left(\frac{k_2}{5k_4}\right)[NaClO_3] = 0.30 \quad \text{for 1.2 M } HClO_4 \quad \text{or} \quad K_{34C} = 0.58 \text{ for 2 M } HClO_4 \qquad \text{(S11)}$$

Initial conditions:

At $t^* = 0$ the concentrations, $y_i$ (i = 5, 4, 3 or 1) are equal to their initial values: $y_i = y_i^0$, in particular, $y_5 = 1$ while $y_4 = y_4^0 = [ClO_2]^0/[NaClO_3] = 1.6\ 10^{-3}$. The other initial values, $y_3^0$ and $y_1^0$, are unknown, even though they are expectedly very small.

Further theoretical analysis based on kinetic equations (S6), (S7), (S8) and (S9) for Schemes A, B, C, BC and KG is presented in sections **S4** and **S5** below. It includes the description of their numerical solution for any set of the values of their dimensionless parameters as well as of the determination of these values of the parameters via comparison of the numerical solutions with experimental data for the time-dependent evolution of the $ClO_2$ concentration (shown in Figure 5a). It has been shown that such a comparison of the calculated (for the optimal values of the fitting parameters) and experimental results is useful to perform with the use of *two different* coordinates: $y_4$ *vs*. $t^*$ and log $y_4$ *vs*. $t^*$. The choice among these coordinates corresponds to the fitting procedure on the basis of different relations for the distance between the calculated and theoretical plots, Equations (S15) and (S17), respectively, see sections **S4** and **S5**.

## S4. Numerical solution of kinetic equations

For each scheme the set consists of 4 coupled ordinary differential equations of the first order of the form:

$$\frac{dy_i}{dt^*} = F_i(y_j;\ j = 5,4,3 \text{ and } 1) \quad \text{for } t^* > 0 \qquad \text{(S12)}$$

with initial conditions at $t^* = 0$: $y_i = y_i^0$ where the initial values, $y_i^0$, are considered *as known numbers* (see below).

There is only one solution of this Cauchy problem which may be found by *numerical integration*. The 4$^{th}$ order Runge-Kutta method has been used in this study for numerical solution of various sets of kinetic equations, section "2.5. Dimensionless variables".

These calculations provide a table containing the values of all dimensionless concentrations, $y_i$ (i = 5, 4, 3 or 1), for the set of temporal points, $t^*_n$.

## S5. Fitting procedure

It is based on comparison of the calculated values of the *dimensional* $ClO_2$ concentration, i.e. values of the $y_4^{(calc)}$-*vs.*-$t^*$ function, for a chosen set of the dimensionless parameters ($K_{54}$, $K_{34}$ of

Schemes A, B or C as well as initial values of the concentrations, $y_i^0$) with experimental data for this function in Figure 5a recalculated for points: $t^*_n = k_4\ t_n$. Namely, for each calculated plot, $y_4^{(calc)}(t^*)$, the mean-square difference, SV, between the calculated, $y_4^{(calc)}{}_n = y_4^{(calc)}(t^*_n)$, and experimental, $y_4^{(exper)}{}_n = y_4^{(exper)}(t^*_n)$, values for a set of dimensionless time moments, $t^*_n$ , is calculated:

$$\mathrm{SV} = \mathrm{N}^{-1}\sum \mathrm{W}_n \left[y_{4,n}^{(calc)} - y_{4,n}^{(exper)}\right]^2 \qquad \text{(S13)}$$

where N + 1 is the number of temporal points (N = 43), $W_n$ is weight factor of the nth point. The summation is carried out for the values of n from 0 to N. The points along the dimensionless time coordinate, $t^*_n$, are distributed in a uniform manner: $t^*_n = n\ \Delta t$, $\Delta t = 0.47915$, $t^*_N = 20.60$.

Analogous parameter in the *dimensional* variables, $SV_{dim}$, defined by the differences between the calculated and experimental values of the dimensional concentrations of $ClO_2$:

$$\mathrm{SV}_{dim} = \mathrm{N}^{-1}\sum \mathrm{W}_n \left(\left[\mathrm{ClO_2}\right]_n^{(calc)} - \left[\mathrm{ClO_2}\right]_n^{(exper)}\right)^2 = \left([\mathrm{NaClO_3}]\right)^2 \mathrm{SV} \qquad \text{(S14)}$$

differs from SV by a constant multiplier, see Equation (40).

A frequent choice of the weight factors is their identical values for all point, i.e. $W_n = 1$ for all points. Then, the mean-square difference, SV, characterizes the *average value* of the difference between the calculated and experimental values of the $ClO_2$ concentration squared:

$$\mathrm{SV}^{abs} = \mathrm{N}^{-1}\sum \left[y_{4,n}^{(calc)} - y_{4,n}^{(exper)}\right]^2 \qquad \text{(S15)}$$

so that this global parameter, $SV^{abs}$, characterizes the average distance between these plots *in the linear coordinates*, *$y_4$ vs. $t^*$*.

Such a calculation gives values of $SV^{abs}$ as a function of all unknown values of parameters. These values of parameters are chosen initially in a random manner, thus giving a starting value of $SV^{abs}$. Then, the values of unknown parameters vary in the way to minimize the mean-square difference, $SV^{abs}$, between the newly calculated and experimental plots. As a result, the procedure determines the *optimal* values of the fitted parameters which correspond to the minimal value of the difference, $SV^{abs}$, for the plots *in the linear coordinates*, *$y$ vs. $t^*$*.

Owing to the general relation between the values of SV and $SV_{dim}$ given by Equation (S14) for any set of the weight factors, $W_n$, the *optimal* values of all fitting parameters (see above) are the same on whether the minimization procedure is performed in the *dimensional* or *dimensionless* variables.

In view of the proportionality between the temporal dependences of the current density and the $ClO_2$ concentration (Figure 5e) the *optimal* values of the fitting parameters are the same for both plots. It is why the fitting will only be carried out for the temporal dependence of the $ClO_2$ concentration (see black points in Figure 6).

Such a choice of the weight factors, $W_n$, see Equation (S15), is well suitable to decide on whether the calculated plot for the temporal evolution of the $ClO_2$ concentration (see e.g. Figure 6a) reproduces properly principal features of the experimental plot, $y_4^{(exper)}$ *vs*. $t^*$.

At the same time there is an important feature of the plots which is *not* immediately visible in the *linear* coordinates. Namely, it is evident that both the calculated and experimental values of the $ClO_2$ concentration are *very small* within the *initial* time range of the process but one *cannot* conclude on the basis of Fig. 6a on whether the calculated plot is able *to reproduce quantitatively* experimental data within this range, *or not*.

It is the reason why it is worth to supplement such an analysis in the *linear* coordinates (Figure A1a) by that in the *semi-logarithmic* ones, i.e. $\log y_4$ *vs*. $t^*$ (see e.g. Figure 6b). This second graph is able to provide an *extra useful information*. First of all, one can judge on whether the theoretical scheme is able to reproduce the behavior of the experimental plot *within the initial stage of the global process* where the $ClO_2$ concentration is still *close to its initial value*, $y_4^0$. Figure 6b gives immediately an *affirmative* answer to this question. Besides, one can see that both the growing and decreasing branches of the experimental plot are *close to straight lines* (with different slopes) within almost whole-time ranges (with relatively small deviations from the linearity), except a relatively narrow time interval in the vicinity of the maximum, but including the *initial* time range. Such a behavior is also a typical feature of *autocatalytic* processes. On the other hand, the time range of relatively *high* $ClO_2$ concentrations where the theoretical plot does *not match well* with the experimental one is evidently *preferably to analyze* in the *linear* coordinates (Figure 6a) since such deviations are *less clearly visible* for the semi-logarithmic coordinates.

The distance between the theoretical and experimental plots in Fig. A1b: could be estimated, e.g. with the use of an analog of Equation (S15):

$$SV^{\log} = N^{-1}\sum\left[\log(y_{4,n}^{(calc)}) - \log(y_{4,n}^{(exper)})\right]^2 = N^{-1}\sum\left[\log\left(\frac{y_{4,n}^{(calc)}}{y_{4,n}^{(exper)}}\right)\right]^2 \quad \text{(S16)}$$

Assuming that the ratios, $y_4^{(calc)}{}_n / y_4^{(exper)}{}_n$, are mostly close to 1, one can use the approximation: $\log (y_4^{(calc)}{}_n / y_4^{(exper)}{}_n) \cong -1 + y_4^{(calc)}{}_n / y_4^{(exper)}{}_n$.

As a result, the value of distance, $SV^{\log}$, is close to the *average value* of the difference between the *relative* values of the calculated and experimental $ClO_2$ concentration squared:

$$SV^{\log} \cong SV^{relative} = N^{-1}\sum\left[1 - \frac{y_{4,n}^{(calc)}}{y_{4,n}^{(exper)}}\right]^2 = N^{-1}\sum\left[\frac{y_{4,n}^{(calc)} - y_{4,n}^{(exper)}}{y_{4,n}^{(exper)}}\right]^2 \quad \text{(S17)}$$

It means that one should use Equation (S17) for the fitting procedure if we want to reach *a uniform proximity within the whole time range* (including the initial time range where the $ClO_2$ concentration is very small compared to the one within the medium and final time intervals).

Thus, the fitting procedure for each kinetic scheme has been carried out with the use of both Equation (S15) and Equation (S17), each of them providing its own merits and disadvantages.
